# The Barley *HvWRKY6* Transcription Factor Is Required for Resistance Against *Pyrenophora teres* f. *teres*

**DOI:** 10.3389/fgene.2020.601500

**Published:** 2021-01-15

**Authors:** Prabin Tamang, Jonathan K. Richards, Shyam Solanki, Gazala Ameen, Roshan Sharma Poudel, Priyanka Deka, Karl Effertz, Shaun J. Clare, Justin Hegstad, Achintya Bezbaruah, Xuehui Li, Richard D. Horsley, Timothy L. Friesen, Robert S. Brueggeman

**Affiliations:** ^1^Department of Plant Pathology, North Dakota State University, Fargo, ND, United States; ^2^Department of Plant Pathology and Crop Physiology, Louisiana State University, Baton Rouge, LA, United States; ^3^Department of Crop and Soil Sciences, Washington State University, Pullman, WA, United States; ^4^Department of Civil and Environmental Engineering, North Dakota State University, Fargo, ND, United States; ^5^Department of Plant Sciences, North Dakota State University, Fargo, ND, United States; ^6^Cereal Crops Research Unit, United States Department of Argiculture – Agricultural Research Service, Edward T. Schafer Agricultural Research Center, Fargo, ND, United States

**Keywords:** barley, disease resistance and susceptibility, *Pyrenophora teres* f. *teres*, net blotch of barley, mutants, exom capture, WRKY transcription factor

## Abstract

Barley is an important cereal crop worldwide because of its use in the brewing and distilling industry. However, adequate supplies of quality malting barley are threatened by global climate change due to drought in some regions and excess precipitation in others, which facilitates epidemics caused by fungal pathogens. The disease net form net blotch caused by the necrotrophic fungal pathogen *Pyrenophora teres* f. *teres* (*Ptt*) has emerged as a global threat to barley production and diverse populations of *Ptt* have shown a capacity to overcome deployed genetic resistances. The barley line CI5791 exhibits remarkably effective resistance to diverse *Ptt* isolates from around the world that maps to two major QTL on chromosomes 3H and 6H. To identify genes involved in this effective resistance, CI5791 seed were γ-irradiated and two mutants, designated CI5791-γ3 and CI5791-γ8, with compromised *Ptt* resistance were identified from an M_2_ population. Phenotyping of CI5791-γ3 and -γ8 × Heartland F_2_ populations showed three resistant to one susceptible segregation ratios and CI5791-γ3 × -γ8 F_1_ individuals were susceptible, thus these independent mutants are in a single allelic gene. Thirty-four homozygous mutant (susceptible) CI5791-γ3 × Heartland F_2_ individuals, representing 68 recombinant gametes, were genotyped via PCR genotype by sequencing. The data were used for single marker regression mapping placing the mutation on chromosome 3H within an approximate 75 cM interval encompassing the 3H CI5791 resistance QTL. Sequencing of the mutants and wild-type (WT) CI5791 genomic DNA following exome capture identified independent mutations of the *HvWRKY6* transcription factor located on chromosome 3H at ∼50.7 cM, within the genetically delimited region. Post transcriptional gene silencing of *HvWRKY6* in barley line CI5791 resulted in *Ptt* susceptibility, confirming that it functions in NFNB resistance, validating it as the gene underlying the mutant phenotypes. Allele analysis and transcript regulation of *HvWRKY6* from resistant and susceptible lines revealed sequence identity and upregulation upon pathogen challenge in all genotypes analyzed, suggesting a conserved transcription factor is involved in the defense against the necrotrophic pathogen. We hypothesize that *HvWRKY6* functions as a conserved signaling component of defense mechanisms that restricts *Ptt* growth in barley.

## Introduction

A recent study determined that climate change is a major threat to malt barley production as yield loss is projected due to high temperatures and water deficiency in some growing regions (Xie et al., 2018). However, as climate change brings drought to some regions, others will experience excess precipitation and combined with elevated temperatures, will provide environments that are more conducive to fungal disease epidemics. Thus, without adequate management tools, disease problems can be expected to be exacerbated in these regions. The threat of greater disease epidemics due to rising temperatures in regions with excess precipitation was not accounted for in the predictions of world barley shortages (Xie et al., 2018), thus production shortfalls could be even greater than predicted. For sustainable barley production, the intelligent deployment of durable genetic resistance to important fungal pathogens is critical and is a major focus of breeding programs with the primary goal of releasing high yielding and broadly adapted varieties that produce quality grain across dynamic environments (Horsley et al., 2009).

The net blotch of barley (*Hordeum vulgare* L.), caused by *Pyrenophora teres* Drechs. is an economically important foliar disease in barley growing regions worldwide with epidemics causing 10–40% of yield loss when susceptible varieties are grown, but under environmental conditions conducive to disease epidemics losses can reach 100% (Mathre, 1997; Murray and Brennan, 2010). Because the brewing and distilling industries demand quality malting barley, which brings premium prices, producers are concerned by biotic or abiotic stresses that negatively affect yield and quality (Grewal et al., 2008). Foliar infection by *P. teres* is a major concern as it has a large impact on yield, but foliar and kernel infection can also impact quality (Liu et al., 2011). The most sustainable and environmentally friendly way to manage net blotch is deploying effective genetic resistance into varieties, yet, a better understanding of this complex pathosystem and the quantitative nature of the host pathogen genetic interactions is needed. Practicing the stewardship of effective resistance sources is important when deploying resistance so that resistance is durable and genes are conserved. One way of accomplishing this goal is through gene discovery and subsequent functional analysis which is more practical with the new array of genomic tools available to the barley research community.

**FIGURE 1:**
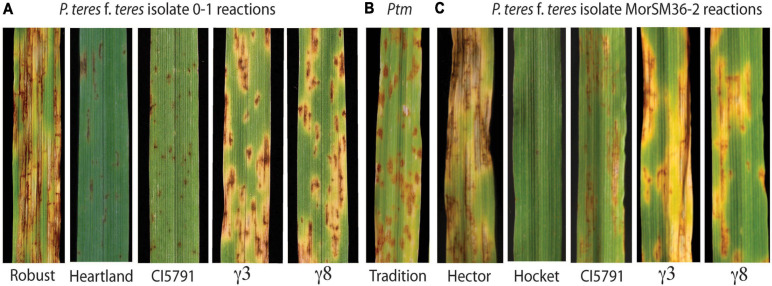
Phenotypic reactions of wild-type CIho 5791, mutants, and resistant and susceptible checks inoculated with *P. teres*. **(A)** Phenotypic reaction of Robust, Heartland, CI5791, CI5791-γ3 (γ3), and CI5791-γ8 (γ8) to *Pyrenophora teres* f. *teres* (*Ptt*) isolate 0-1. **(B)** Phenotypic reaction of the barley cultivar Tradition to *P. teres* f. *maculata* (*Ptm*) isolate FGOB10Ptm-1. **(C)** Phenotypic reaction of Hector, Hockett, CI5791, CI5791-γ3 (γ3), and CI5791-γ8 (γ8) to *Ptt* isolate MorSM36-2. The disease was scored based on a 1–10 rating scale with 1 being highly resistant and 10 being highly susceptible.

Net blotch exists in two forms; net form net blotch (NFNB) caused by *Pyrenophora teres* f. *teres* (*Ptt*) and spot form net blotch (SFNB) caused by *Pyrenophora teres* f. *maculata* (*Ptm*) (Smedegård-Petersen, 1971; Steffenson and Webster, 1992). The symptoms of NFNB first appear as small dark brown necrotic lesions that expand over time forming net like longitudinal and transverse striated necrotic lesions commonly surrounded by chlorosis on susceptible host genotypes (Figure 1A). The symptoms of SFNB also initially appear as small dark brown necrotic lesions that expand over time producing elliptical necrotic lesions typically surrounded by chlorosis on susceptible host genotypes (Figure 1B). Although, these two pathogens are morphologically identical (conidia and mycelium), their genetics as well as host-pathogen interactions are considered to be relatively distinct (Liu et al., 2011), thus, are considered to be different diseases. However, contrary to this statement the recent review of barley-*Pyrenophora teres* genetic interactions showed that 17 of the 19 *Ptm* resistance/susceptibility loci known to date overlap with *Ptt* resistance/susceptibility loci (Clare et al., 2020). This may include the 3H QTL reported by Koladia et al. (2017), where they identified the *HvWRKY6* transcription factor required for NFNB resistance as there were also SFNB resistance QTL identified by biparental and association mapping within the region. Interestingly, we also generated data showing that the *HvWRKY6* gene described here is also required for *Ptm* resistance (data not presented).

The barley line CIho 5791 (hereafter referred to as CI5791), is an Ethiopian breeding line, that is highly resistant to most *Ptt* isolates collected from barley growing regions worldwide (Mode and Schaller, 1958; Steffenson and Webster, 1992; Wu et al., 2003; Richards et al., 2016; Koladia et al., 2017). CI5791 consistently exhibits a highly resistant reaction at the seedling stage (secondary leaf) mainly consisting of small pinpoint necrotic lesions (Figure 1A) and the resistance translates to the field at the adult plant stages. However, CI5791 resistance has been compromised by Canadian, French (Arabi et al., 1992; Akhavan et al., 2016), and Moroccan isolates that are moderately virulent on CI5791 (Figure 1C). Although, the dominant CI5791 resistance located on chromosome 6H is remarkably broad and effective, it is apparent that pathogen populations have the diversity to overcome this resistance.

Several studies mapped dominant and recessive NFNB resistance genes with different specificities to the centromeric region of barley chromosome 6H (Abu Qamar et al., 2008; Liu et al., 2015; Richards et al., 2016, 2017; Koladia et al., 2017), thus, this important NFNB resistance locus is considered complex, possibly harboring multiple dominant resistance genes and recessive susceptibility genes (Steffenson et al., 1996; Cakir et al., 2003; Wu et al., 2003; Friesen et al., 2006; Koladia et al., 2017). Koladia et al. (2017) also mapped two dominant resistance QTL contributed by CI5791 on chromosome 3H and 6H using a CI5791 × Tifang recombinant inbred line (RIL) population individually inoculated with nine geographically distinct *Ptt* isolates. The major CI5791 6H QTL was shown to be dominantly resistant and was effective against all isolates used in the study. The gene underlying the CI5791 3H QTL was also dominant in nature, but was only effective against two Japanese *Ptt* isolates, JPT0101 and JPT9901. Interestingly, a similar chromosome 3H QTL was also contributed by Tifang and was shown to confer dominant resistance against *Ptt* isolates Br. Pteres (Brazil), BB06 (Denmark), 6A (California, United States), and 15A (California, United States).

Exome capture is a cost-effective yet powerful genomics tool that allows for targeted sequencing of the coding regions (exons) of specific specie genomes. This tool has been used for the identification of polymorphism within coding regions that contribute to disease in humans, other animals, and plants (Choi et al., 2009; Raca et al., 2010; Wang et al., 2010; Bamshad et al., 2011; Cosart et al., 2011; Mascher et al., 2013b, 2014, 2016; Warr et al., 2015; Russell et al., 2016). The exome capture array specific to barley (*Hordeum vulgare*), representing 61.6 mega bases of coding region from the complex ∼5.1 Gb barley genome, is available (Mascher et al., 2013b). This array was used to study the domestication and evolution of barley by resequencing and identifying variants in wild barley (*Hordeum sponteneum*) and land race (*Hordeum vulgare*) coding regions, including ancient barley germplasm (Mascher et al., 2016; Russell et al., 2016). Mascher et al. (2014) also identified the barley *HvMND* gene that governs increased tiller numbers utilizing this array. Schreiber et al. (2019) utilized the array on a highly mutagenized TILLING population of the barley variety Golden Promise to identify and evaluate mutation density. They also assembled a collection of semi-sterile mutants from the population and developed a custom exome capture array of 46 candidate genes to identify potential mutations causing the sterility phenotype (Schreiber et al., 2019). The exome capture methodology has also been applied to resistance gene enrichment sequencing (RenSeq), single-molecule real-time RenSeq (SMRT RenSeq), mutagenesis RenSeq (MutRenSeq), and association genetics (AgRenSeq) technology designed to identify nucleotide binding site-leucine rich repeat (NLR) disease resistance genes (*R* genes) in plants (Jupe et al., 2013; Steuernagel et al., 2016; Witek et al., 2016; Arora et al., 2019). A cDNA RenSeq method was also utilized to accelerate the identification of *R* genes in tomato (Andolfo et al., 2014).

The exome capture method coupled with forward genetics screens was considered an efficient genomics tool for the identification of resistance/susceptibility genes. Thus, we utilized this methodology to efficiently identify a gene required for broad and effective CI5791 NFNB resistance underlying the major QTL located on chromosomes 3H and 6H previously identified by Koladia et al. (2017). A CI5791 γ-irradiated mutant population was created and screened for individuals with compromised *Ptt* resistance. Utilizing a forward genetics screen, exome capture, and comparative sequence analysis, the *HvWRKY6* transcription factor (TF) gene underlying the CI5791 chromosome 3H *Ptt* resistance QTL was identified as being required for broad *Ptt* resistance. To the best of our knowledge this is the first gene identified that contributes to NFNB resistance in barley.

## Materials and Methods

### Mutants Development

The Ethiopian barley line CI5791 is highly resistant to most NFNB isolates collected worldwide. A g-irradiation approach was used to develop a CI5791 mutant population. Briefly, ∼500 g of seed was hydrated in an airtight container with 60% glycerol for about 7–10 days. The hydrated seeds were irradiated with 35 kilorads (350 Gy) of g rays in a Gammator (M38-4, Radiation Machinery Corporation) prior to planting. Approximately, 1,400 M_1_ seeds were planted in trays and allowed to self-generate the M_2_ generation. Approximately 10,000 M_2_ seedlings, derived from the original 1,400 M_1_ individuals were screened by inoculation with the *Ptt* isolate LDNH04Ptt19 (hereafter referred to as LDN) collected from Langdon, North Dakota. Planting, inoculum preparation, inoculation, and disease evaluations were performed as described in Friesen et al. (2006). After identifying putative mutants, these seedlings were transplanted to 15.24 cm (6 inch) pots and allowed to self-generate M_3_ generation seeds. The M_3_ generation seeds were planted in cone-tainers and screened with *Ptt* isolate 0-1 using three replicates with WT CI5791 and Heartland as the resistant checks and the susceptible check Robust. *Ptt* isolate 0-1 is a Canadian isolate collected from Ontario (Weiland et al., 1999; Wyatt et al., 2018) and has similar virulence as the isolate LDN which was used to identify the original CI5791-γ3 and CI5791-γ8 mutants from the M_2_ generation. The planting, inoculum preparation, inoculation, and disease reading were performed as described in Friesen et al. (2006).

### Mapping Populations and Phenotyping

Two F_2_ mapping populations were developed by crossing CI5791-γ3 and CI5791-γ8 homozygous mutant M_3_ individuals with the NFNB resistant barley line Heartland. Heartland is a six-rowed spring feed barley that was developed at the Agriculture Canada Research Station, Brandon, Manitoba and registered and released in 1984 (Therrien and Wolfe, 1985). Heartland was shown to be resistant to three major Canadian races of *Ptt* before its release. Heartland is hypothesized to contain a similar dominant resistance gene as CI5791 at the chromosome 6H locus designated as *Rpt5*. The planting, inoculum preparation, inoculation, and disease reading for the F_2_ individuals from each of the CI5791-γ3 and CI5791-γ8 × Heartland populations were performed as described in Friesen et al. (2006). The disease reading was performed 7 days after inoculation (DAI) using a 1–5 rating scale (Neupane et al., 2015) for CI5791-γ3 × Heartland due to the phenotypic resemblance to *Ptm* infection and a 1-10 rating scale developed by Tekauz (1985) for CI5791-γ8 × Heartland. The CI5791-γ3 × Heartland F_2_ susceptible individuals using a rating cutoff of > 2 representing the homozygous CI5791 genotype at the mutant region were used for mapping the gene. We utilized PCR genotyping by sequencing (PCR-GBS) to genotype CI5791-γ3 × Heartland F_2_ homozygous susceptible lines (a total of 34 lines representing 68 recombinant gametes) on an Ion Torrent^TM^ PGM: a PCR-GBS marker panel designed for polymorphism between CV. Tradition and barley line PI67381 consisting of 365 markers (Supplementary Table 1) was used to genotype all 34 susceptible F_2_ lines. Primer development, DNA extraction, PCR cycle parameters, library preparation, and sequencing on the Ion Torrent^TM^ PGM were performed as previously described in Richards et al. (2016).

The disease severity of the 34 CI5791-γ3 × Heartland F_2_ homozygous susceptible lines along with the genotypic data were used for QTL mapping using MapDisto 2.0 (Heffelfinger et al., 2017) and QGene 4.0 (Joehanes and Nelson, 2008). The individual SNP calls were filtered for a minimum genotype quality of 10, and a minimum read depth of 3. The markers with more than 30% missing data and MAF < 25% were removed from further analysis. Single marker regression was used to identify the susceptible QTL in the γ3 × Heartland F_2_ population. CI5791 and the two mutants were also phenotyped with the two Moroccan *Ptt* isolates SM36-2 and SM36-3 that were shown to be moderately virulent on CI5791. The spot form net blotch susceptible barley line Tradition was inoculated with *Ptm* isolate FGOB10Ptm-1 as previously described in Neupane et al. (2015) to compare a typical SFNB phenotype with the NFNB phenotypes on the CI5791-γ3 and CI5791-γ8 mutants (Figure 1).

### Allelism Tests

Reciprocal crosses were made between CI5791-γ3 and CI5791-γ8 to determine if the two putative independent mutants were allelic. Six F_1_s of CI5791-γ3 × CI5791-γ8 and ten F_1_s of CI5791-γ8 × CI5791-γ3 were phenotyped as previously described using the 1–10 rating scale developed by Tekauz (1985) with *Ptt* isolate 0–1. The F_1_ individuals from both crosses were also genotyped utilizing the STS markers targeting the putative mutant gene underlying the region delimited by genetic mapping. The primers used for STS marker development are described below under the STS marker development and mutation validation section.

### Exome Capture Sequencing and Analysis

**FIGURE 2:**
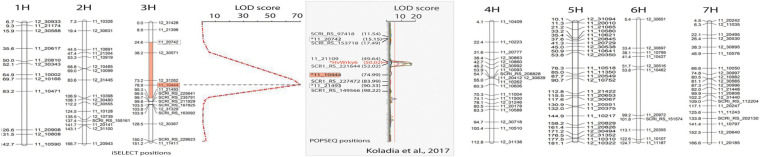
Linkage map developed using 34 CI5791-γ3 × Heartland F_2_ susceptible individuals (representing 68 recombinant gametes) developed with 123 polymorphic SNP markers showing the seven barley chromosomes. For chromosome 3, the QTL map of resistance/susceptibility to *Pyrenophora teres* f. *teres* isolate 0–1 using single marker regression analysis showing the only significant peak (red dashed line) with a LOD score of 71. The *X*-axis represents LOD values and the *Y*-axis represents the PCR-GBS SNP markers. The most significant marker was 11_10444 (red boxes) positioned at 78.9 cM with iSelect positions used to develop the map and 74.99 cM with POPSEQ positions with a LOD value of 71. The red region filled in on the chromosome 3 map flanked by the markers 11_20742 and 11_21493 (white boxes) shows the high confidence interval region containing the CI5791-γ3 mutation. The gray box shows the comparative region mapped by Koladia et al. (2017) with the flanking (white boxes) and most significant (red box) markers from our mapping shown relative to their map. The *HvWRKY6* gene is shown within the QTL detected in their CI5791 × Tifang biparental mapping population. Markers and the *HvWRKY6* gene marked with an asterisk were not mapped by Koladia et al. (2017) but were placed on the map based on their POPSEQ positions for comparison. The markers without an asterisk were mapped by Koladia et al. (2017) but were given POPSEQ positions.

**FIGURE 3:**
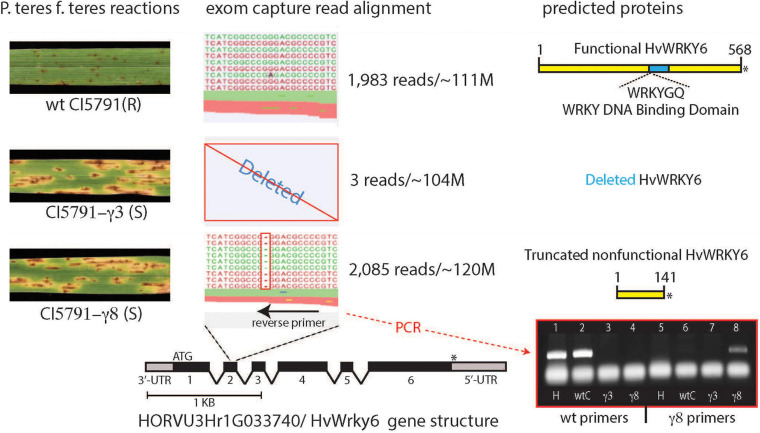
Alignment of exome capture reads from wild-type (WT) CI5791, and the CI5791-γ3 and CI5791-γ8 mutants to the HORVU3Hr1G033740.2 (*HvWRKY6*) gene model from the cultivar Morex genome sequence. The left shows the reaction of WT CI5791, CI5791-γ3, and CI5791-γ8 to *Pyrenohpora teres* f. *teres* isolate 0-1. The center shows the pile up of reads from each genotype to the HORVU3Hr1G033740.2 (*HvWRKY6*) gene model. The alignment shows only three reads in CI5791-γ3, and ∼2,000 reads in both CI5791-γ8 and WT CI5791 confirming the complete gene deletion in CI5791-γ3. The CI5791-γ8 pileup shows a single bp deletion in the 2nd exon. Reverse PCR primers were developed from the deletion region that produced WT specific amplicons and CI5791-γ8 specific amplicons as shown in the bottom right. The WT specific primers amplified only from Heartland and WT CI5791. The mutant specific primers amplified only from CI5791-γ8 and no amplification in CI5791-γ3 as it is missing the entire gene sequence. The right side shows the predicted proteins encoded by the *HvWRKY6* gene in each genotype.

The exome capture, sequencing, and analysis of the CI5791-γ3 and CI5791-γ8 mutants and CI5791 WT were performed as thoroughly described in Solanki et al. (2019). The POPSEQ positions of the markers flanking the QTL identified in the segregating F_2_ population (Figure 2), described above, were obtained and used to identify exome capture targets within the mapped region. BAM files from the analysis were imported into CLC Genomics Workbench version 8.0.3 (Qiagen) for the visualization of sequence alignments (Figure 3).

### STS Marker Development and Mutation Validation

Based on the identified nucleotide deletion in the CI5791-γ8 mutant specific sequence tagged site (STS), markers was developed. Oligonucleotide primer pairs were developed specific to CI5791 WT and the CI5791-γ8 mutants. Primers WRKY6-F1 (5′-GCCGCTGGTTCTCGTCG TTCATGCG-3′) and WRKY6-Wt-R1 (5′-TAGTCGACGACGACGGGGCGTCCC-3′) only produced an amplicon from CI5791 WT (Figure 3) whereas the primer combination of WRKY6-F1 and WRKY6-Mt-R2 (5′-TAGTCGACGACGACGGGGCGT CCG-3′) only produced an amplicon from the CI5791-γ8 mutant due to designing specificity in the 3 bases at the 3′ terminus of the primer that are specific to the 1 bp deletion discovered in the CI5791-γ8 mutant from the exome capture data. The PCR was optimized so the discriminant amplicons were specific to the WT or mutant genotypes. The PCR amplification program was set as: denaturation at 95^o^C for 5 min, 25 cycles of 95^o^C for 30 s, 76^o^C for 1 min, and 76^o^C for 30 s, and a final extension of 72^o^C for 5 min. Wild-type CI5791, Heartland, CI5791-γ3, CI5791-γ8, homozygous susceptible F_2_ individuals from two populations, and 15 randomly selected resistance F_2_ lines (CI5791-γ8 × Heartland) were genotyped with the WT and mutant specific primers. The F_1_ reciprocal cross between CI5791-γ3 and CI5791-γ8 were also genotyped with these primer sets. All PCR amplicons were visualized on a 1% agarose gel with GelRed^®^ (Biotium).

### HvWRKY6 Allele Sequencing and Analysis

To compare if there was the presence of any allelic variation between resistant and susceptible barley cultivars, we sequenced *HvWRKY6* from CI5791 (resistant), cvs. Tifang (susceptible), and Morex (moderately susceptible). We designed four primer pairs at 1 kb intervals to sequence the entire gene including the promoter region (∼3,544 bp) (Supplementary Table 2). The gDNA extractions were performed as previously described in the “DNA Extraction, Exome Capture, and Sequencing” section above and were quantified using the Qubit^TM^ 2.0 Fluorometer with a Qubit^TM^ dsDNA Broad Sensitivity Kit (Thermo Fisher Scientific). PCR parameters were initial denaturation at 95°C for 5 min, 35 cycles of 94°C for 30 s, 60°C for 60 s, and 72°C for 60 s, followed by a final extension at 72°C for 5 min. PCR amplicons were visualized on a 1% agarose gel containing GelRed^®^ (Biotium) and purified using an E.Z.N.A.^®^ Cycle Pure centrifugation columns (Omega Bio-tek) following the manufacturer’s protocol. Purified PCR products, ∼40 ng, were sent to GenScript for sequencing following their guidelines.

### RNA Extraction, cDNA Synthesis, and qPCR

**FIGURE 4:**
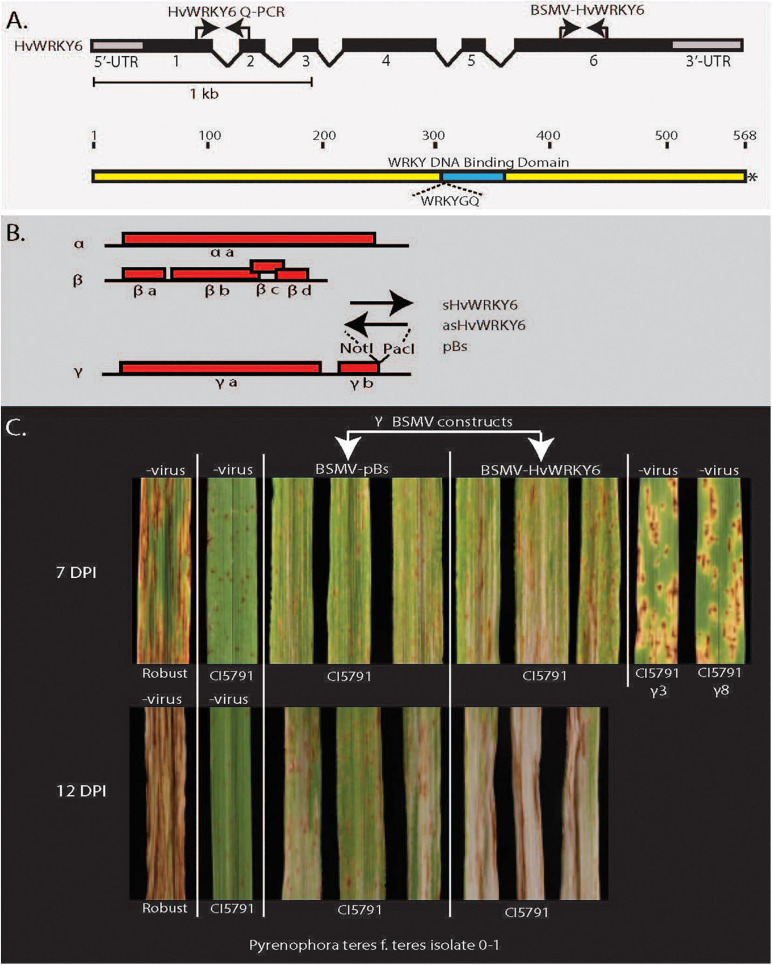
Barley stripe mosaic virus-virus induces gene-silencing (BSMV-VIGS) validation of *HvWRKY6* function in *Pyrenophora teres* f. *teres* resistance in barley line CI5791. **(A)** The *HvWRKY6* transcription factor gene and protein structure showing location of primers used for qPCR and BSMV-VIGS constructs. Black bars at the top represent the intron/exon structure of the *HvWRKY6* gene with black bars indicating exons and the gray terminal bars indicating the 5′ and 3′ untranslated regions (UTRs). **(B)** The red bars below show the barley stripe mosaic virus genomic RNA with red boxes indicating viral genes encoded from the positive stranded RNA virus genome. The black arrows indicate where the sense and antisense *HvWRKY6* fragments were inserted into the γ genome at the *Not*I and *Pac*I restriction sites of the infectious BSMV *E. coli* plasmid pSL38.1 to develop *HvWRKY6* post transcriptional gene silencing constructs. **(C)** The BSMV-VIGS experiments showing that the specific silencing of the candidate *HvWRKY6* gene results in susceptible reactions when inoculated with *P. teres* f. *teres* isolate 0–1 at 7 and 12 days post inoculation. The BSMV-VIGS pBs vector control does not show the shift from resistance toward susceptibility.

Quantitative PCR (qPCR) was conducted to measure if differential regulation of the *HvWRKY6* gene occurred upon interaction with *Ptt* isolates in compatible or incompatible interactions. The isolates *Ptt* 0-1, SM36-2, and SM36-3 were used to inoculate CI5791 and the barley line Tifang was inoculated with *Ptt* 0-1 only. Primers were designed across exons 1 and exon 2 (Figure 4): wrky6-qpcr-F2 (5′-GTTCCTGCCGTTACTGTCCTCATC-3′) and wrky6-qpcr-R2 (5′-TCGCCATCAAGAAGGAGGACCTCAC-3′), that specifically amplify ∼120 bp from cDNA and ∼270 bp from gDNA. At least three biological replications were collected from each mock (water + tween 20) and *Ptt* inoculated plants. Tissues from the first leaves were collected at time point 0 (non-inoculated control), 5, 30 min, 1, 2, 4, 6, 12, 24, 48, 72, 96, 120, 144, and 168 h post inoculation. Tissue samples were immediately flash frozen in liquid nitrogen and stored at −80°C for further processing. Total RNA was extracted from the collected tissue using an RNeasy Plant Mini Kit (Qiagen) following the manufacturer’s instructions. The total RNA was quantified using the Qubit^TM^ Fluorometer and the Qubit^TM^ RNA BR assay kit (Thermo Fisher Scientifics) per the manufacturer’s instructions. To ensure RNA integrity and that the RNA was free of gDNA contamination, 1 μl of total RNA was denatured in 4 volumes of denaturing buffer (Formaldehyde Load Dye, Ambion) at 80°C for 5 min and visualized on a 1% agarose gel with GelRed^®^ (Biotium). RNA samples with the four-intact ribosomal RNA (rRNA) bands at the expected molecular weights of ∼ 3.4, 1.8, 1.5, and 1.1 kb corresponding to the nuclear 28S and 18S rRNAs and the 23S and 16S plastid rRNAs, respectively, without high molecular weight gDNA contamination were considered as quality RNA and used for cDNA synthesis. The GoScript^TM^ Reverse Transcription System (Promega) was used to synthesize cDNA following the manufacturer’s protocol. Briefly, ∼1 μg of total RNA was mixed with oligo(dT)_15_ primer (0.5 μg) and incubated at 70°C for 5 min. The RNA sample was then mixed with 15 μl of reverse transcription reaction mix (GoScript^TM^ Reaction Buffer (5X), MgCl_2_ (1.5 mM), PCR Nucleotide Mix (0.5 mM each dNTP), Recombinant RNasin Ribonuclease Inhibitor (20 units), and Reverse Transcriptase, and incubated at 25°C for 5 min followed by 42°C for 60 min and inactivated at 70°C for 15 min. The 20 μl cDNA synthesis reactions were diluted with 80 μl H_2_O (1:5). A 10 μl qPCR reaction was prepared by mixing 4 μl of diluted cDNA, 5 μl of SsoAdvanced Universal SYBR Green Supermix (Bio-Rad), and 0.5 μl of each forward and reverse primer (10 μM). The qPCR was conducted in a CFX96 Real-time system thermocycler (Bio-Rad) with cycling parameters of 95°C for 30 s followed by 40 cycles of 95°C for 15 s and 60°C for 30 s; 65°C for 30 s; and 60 cycles of temperature increasing from 60 to 95°C with fluorescence readings acquired at 0.5°C increments per cycle. Three technical replications were used for each biological rep. The barley *HvSnor14* gene was used as the reference (Ferdous et al., 2015) to normalize *HvWRKY6* gene expression. The efficiency of qPCR for the *HvWRKY6* and S*nor14* primers were calculated by generating a standard curve by running qPCR on a 10-fold serial dilution starting from 200 pg of the PCR amplified template of *HvWRKY6* and *Snor14*. Differential expression was calculated by using the ΔΔCT method on the Bio-Rad CFX Manager 3.1 software. A *t*-test was performed to check the significance of difference at *p* < 0.05 using a standard error of mean of 1.

### BSMV-VIGS

The barley stripe mosaic virus-virus induced gene silencing (BSMV-VIGS) system was exploited to functionally validate the *HvWRKY6* as required for resistance in the barley line CI5791. A unique 65 bp sequence was selected from the *HvWRKY6* gene by performing a BLASTn search against the low and high confidence gene list in the IPK barley database^1^ to reduce the cross amplification and off target silencing of other WRKY TF homologs in the barley genome. Two primer pairs based on the 5′ and 3′ termini of this unique sequence were designed with *Not*I and *Pac*I adaptor sequences attached to the 5′ ends of the respective primers. These adaptors were reciprocally utilized in order to develop sense and antisense constructs. The first primer set was designed with a *Not*I adaptor on the forward primer and *Pac*I adaptor on the reverse primer and the second set with the *Pac*I adaptor on the forward primer and *Not*I adaptor on the reverse primer.

#### First Primer Set

WRKY6_KD_NtFP1- GGAGCGGCCGCACGCCATGCC GCTAAACGTCG
WRKY6_KD_PcRP1- GGATTAATTAAGCCGGGCATC GGAACATGGAAC

#### Second Primer Set

WRKY6_KD_PcFP1-GGATTAATTAAACGCCATGCC GCTAAACGTCG
WRKY6_KD_NtRP1-GGAGCGGCCGCGCCGGGCAT CGGAACATGGAAC

These two primer sets were used to clone the unique 65 bp *HvWRKY6* fragment into the γRNA strand of the BSMV-VIGS infectious cDNA clone PSL38.1 in both sense and antisense orientations. First, the two primer sets were used to produce the gene specific amplicon from CI5791 cDNA in 20 μl PCR reactions consisting of 2 μl of cDNA template, 0.5 μl of each forward and reverse primers (10 μM), 0.3 μl of dNTPs (500 μM), 0.2 μl of GoTaq^®^ (1.25 units), 4 μl of GoTaq^®^ buffer (10x), and 12.5 μl of H_2_O. The PCR cycle parameters had an initial denaturation at 95°C for 5 min, followed by 35 cycles of 95°C for 30 s, 60°C for 30 s, and 72°C for 30 s followed by a final extension of 72°C for 5 min. The amplicon was purified using an E.Z.N.A^®^ Cycle Pure centrifugation column (Omega Bio-tek). The purified PCR products was digested in a 30 μl reaction consisting of 0.5 μl of *Not*I HF (NEB), 0.5 μl of *Pac*I (NEB), 3 μl of Cut Smart Buffer (NEB), 11 μl of H_2_O, and 15 μl of PCR product. The digestion reaction was allowed to incubate at 37°C for 2 h followed by inactivation at 65°C for 20 min. The BSMV vector PSL38.1-MCS for cloning the target amplicon was also digested with 3 units of *Not*I and *Pac*I double digestion reactions using 5 μg of plasmid in a 30 μl reaction. Digested PCR product (2 μl) was mixed thoroughly in an 8 μl ligation reaction mix comprised of 1 μl of predigested vector (∼80 ng), 1 μl of ligation buffer (10X), 1 μl of T4 DNA ligase, and 5 μl of H_2_O and incubated at 4°C for 24 h. Chemically competent One Shot^®^ TOP10 *E. coli* cells (Thermo Fisher Scientific) according to the manufacturer’s protocol were then transformed with the ligation mix and inoculated into 250 μl of Luria Broth (LB) liquid media and incubated at 37°C with 230 rpm shaking for 1 h. A total of 100 μl of each transformation was plated onto LB agar plates with 100 μg/ml of ampicillin and incubated overnight (∼12 h) at 35°C. Ten random colonies were picked from each transformation and inoculated into 2 ml of LB broth [5 g of NaCl, 5 g of tryptone, 2.5 g of yeast extract, and 500 ml of H_2_O and ampicillin (100 μg/ml)] in 12 ml borosilicate culture tubes and incubated overnight with shaking at 230 rpm at 37°C. The cell cultures were transferred to a 2-ml microcentrifuge tube and centrifuged at 12,000 rcfs for 5 min to pellet the cells and the waste supernatant was discarded. The plasmid DNA was extracted from the pelleted cells using the PureYield^TM^ Plasmid miniprep System (Promega) following the manufacturer’s protocol.

The BSMV tripartite viral genomic RNAs (α, β, and γ genomes) with the γ fragment containing the unique 65 bp fragments of the CI5791 *HvWRKY6* allele cloned in both the sense and antisense orientations, were synthesized via *in vitro* transcription using the mMESSAGE mMACHINE^TM^ T7 Transcription Kit (Thermo Fisher Scientific) according to the manufacturer’s protocol. Twenty microliter reactions of each of the α genome, β genome, γ-*HvWRKY6* sense, and γ-*HvWRKY6* antisense genomes were combined with 370 μl of FES buffer (100 ml of GP buffer, 5 g of sodium pyrophosphate decahydrate, 5 g of bentonite, 5 g of celite, and up to 500 ml of H_2_O) as the BSMV-VIGS inoculum. A total of 20 μl of each of the α genome, β genome, and γ genome were combined with 390 μl FES buffer for the BSMV-VIGS control inoculum.

Single seeds of the barley line CI5791 were planted per cone-tainer and placed in racks. Newly emerged secondary leaves still whorled at the ∼10–11 days old seedling stage were inoculated with either 5 μl of each tripartite RNAs or BSMV-VIGS control virus (both in FES buffer). Approximately 40 individual plants were inoculated for each BMSV-VIGs experimental RNAs and control RNAs. Plants were first misted heavily and then inoculated by gently rubbing the leaves with 5 μl of each BSMV-VIGs construct. After incubation in the mist chamber for 24 h at 100% humidity, inoculated plants were moved back to the growth chamber set at 21°C with a 12 h photoperiod. Once typical BSMV symptoms, mottling and striping, appeared on the expanded or expanding tertiary leaves, plants were inoculated with *Ptt* isolate 0–1 as previously described in Friesen et al. (2006). Inoculum preparation, inoculation, and disease reading were performed as described before. CI5791 and Robust were used as a resistance and a susceptible check, respectively. Disease reading was performed 7 and 12 days after *Ptt* inoculation (Figure 4 and Supplementary Tables 3, 4) using the 1–10 scale developed by Tekauz (1985).

### mRNA Extraction, RNAseq Library Preparation, and Sequencing

Three leaf samples of equal size (∼2 cm) from each replicate per treatment, non-inoculates and inoculated with *Ptt* isolate LDN at 3, 21, and 45 h post pathogen inoculation (hpi), were combined in a single tube and flash frozen in liquid nitrogen then stored at −80°C until further processing for total RNA extraction. The total RNA was extracted from the frozen leaf samples using the RNeasy mini kit (Qiagen) following the manufacturer’s standard protocol. RNA concentrations were measured using the Qubit^®^ Broad Range RNA kit on a Qubit^TM^ 2.0 Fluorometer (Thermo Fisher Scientific), and RNA samples were visualized on 1% agarose gels stained with GelRed^®^ (Biotium) to confirm the integrity of the RNA samples. RNA samples with four sharp ribosomal RNA (rRNA) bands; approximate molecular weights of 3.4, 1.8, 1.5, and 1.1 kb corresponding to nuclear 28S and 18S rRNAs and 23S and 16S plastid rRNAs, respectively, without a high molecular weight genomic DNA contamination band were considered quality RNA. One microgram of total RNA was used for RNA sequencing (RNAseq). The library construction was performed using the TruSeq RNA Library Prep Kit v2 (Illumina) following the manufacturer’s standard protocol. The final library was validated and quantified on the Agilent 2100 Bioanalyzer. The cDNA libraries from four different samples were pooled into one single tube and were normalized according to the manufacturer’s protocol. Each of the library pools were diluted to a concentration of 1.8 pm and sequenced on the Illumina NextSeq^®^ 500 sequencer on a single flow cell at the USDA Cereal Genotyping Centre, Fargo, ND, United States. The NextSeq^®^ 500/550 High Output Kit v2 (150 cycles) was used for the generation of 150 bp single end reads. The raw sequence reads were demultiplexed and converted into individual FASTQ files using bcl2fastq software v2.17.1.14 (Illumina). The FASTQ reads were quality trimmed in CLC Genomics Workbench v8.0.3 (Qiagen) using default settings.

### Expression Analysis

The analysis pipeline for mapping reads to the reference genome, quality check, and for expression analyses was performed by first mapping the high quality trimmed sequencing reads to the barley RefSeq v1.0 (see text footnote 1) in CLC Genomics Workbench v8.0.3 (Qiagen). Gene specific and transcript specific reads were obtained from reference genes as well as from the gene track and mRNA tract information. This enabled reads to align to both intronic and intergenic regions. Reads less than 90% identical for 90% of the read length and that mapped to more than 10 positions were discarded. The total reads mapped for each gene model were normalized to obtain reads per kilobase of exon model per million mapped reads (RPKM) values for each sample. In all the comparisons, the false discovery rate (FDR)-corrected *p*-values were calculated by the exact test using the EdgeR bioconductor package in CLC Genomics Workbench. Analyses were based on a threshold of 0.05 for FDR-corrected *p*-value and a fold change of 3. All treatments were compared with 0 h control (no *Ptt* inoculation).

## Results

### Mutant Identification and Validation

To identify mutants compromised for *Ptt* resistance seeds of the highly resistant barley line CI5791 was γ-irradiated and ∼10,000 M_2_ seedlings derived from ∼1,400 M_1_ individuals were used in a forward genetics screen for their reaction to *Ptt* isolate LDN. Two mutant individuals designated CI5791-γ3 and CI5791-γ8 were identified and advanced to the M_3_ generation. The two mutant lines were confirmed by phenotyping M_3_ individuals in replicated trials and shown to express similar phenotypes resembling susceptible SFNB reactions when inoculated with *Ptt* isolate 0–1 the causal agent of NFNB (Figures 1A,B). The CI5791 WT and the resistant variety Heartland, which was used to develop the mutant mapping populations described below, exhibited highly resistant reactions (pin point necrotic lesions) to *Ptt* isolate 0–1 with an average infection type (IT) of 1.5 based on the 1–10 NFNB rating scale developed by Tekauz (1985) (Figure 1A and Supplementary Tables 1, 5). Phenotyping CI5791-γ3 and CI5791-γ8 showed average ITs of 6.5 and 6.0, respectively with *Ptt* isolate 0–1 using the NFNB 1–10 scale (Tekauz, 1985; Figure 1A and Supplementary Table 5). The susceptible barley variety Robust exhibited typical NFNB susceptible infection types (Figure 1A). Inoculation of CI5791 WT with the Moroccan *Ptt* isolates SM36-2 and SM36-3 exhibited moderately susceptible ITs averaging 4.5 and 3.5, but the mutants were susceptible showing average disease scores of 6.5 and 6 for CI5791-γ3 and 7.0 and 6.5 for CI5791-γ8, respectively (Figure 1C and Supplementary Table 6). The variety Hockett, exhibited highly resistant reactions to *Ptt* isolates SM36-2 and SM36-3, with average ITs of 1 (Figure 1C and Supplementary Table 6). The susceptible barley variety Hector exhibited highly susceptible ITs with an average disease score of 9.5 and 8.0 to isolates SM36-2 and SM36-3, respectively (Figure 1C and Supplementary Table 6).

CI5791-γ3 and CI5791-γ8 were crossed with cultivar Heartland and F_1_ individuals were allowed to self-pollinate to produce the F_2_ populations. The CI5791-γ8 × Heartland F_2_ populations contained 116 individuals. The F_2_ population was inoculated with *Ptt* isolate 0–1 and an IT of 4 was used as the cutoff for resistance/susceptibility with the CI5791-γ8 × Heartland F_2_s on the 1–10 NFNB scale (Tekauz, 1985). The F_2_ population showed a segregation ratio not significantly different from a 3 resistant: 1 susceptible ratio as would be expected for a single recessive mutant gene (Supplementary Tables 1, 5, 7). Since the F_2_ phenotyping data determined that a single recessive mutation was responsible for the susceptible phenotype in the CI5791-γ8 mutant, reciprocal crosses between CI5791-γ3 and -γ8 were made to determine if the mutations were allelic. Six CI5791-γ3 × -γ8 and ten CI5791-γ8 × -γ3 F_1_ individuals were challenged with *Ptt* isolate 0–1 and all the F_1_ individuals showed susceptible reactions that were similar to each of the mutant parental lines with average scores of 6.45 and 6.25, respectively (Supplementary Table 3).

### Mutant Mapping

Thirty-four F_2_ individuals from a CI5791-γ3 × Heartland population showing the characteristic SFNB-like susceptible lesions indicating that they were homozygous for the mutated gene, were genotyped using PCR-GBS to identify 123 polymorphic SNP markers spread across the seven barley chromosomes (Figure 2). The QTL mapping utilizing the genotyping of the 34 CI5791-γ3 × Heartland F_2_ homozygous susceptible lines, representing 68 recombinant gametes, localized the mutation to chromosome 3H within a high confidence ∼75 cM interval flanked by the SNP markers 11_20742 (POPSEQ position; chr = 3H cM = 15.15) and 11_21493 (POPSEQ position; chr = 3H cM = 90.33) (Figure 2). The most significant marker 11_10444 (POPSEQ position; chr = 3H cM = 74.99) had a LOD score of 71 (Figure 2).

### Exome Capture Sequencing and Analysis

Sequencing of CI5791 WT, CI5791-γ3, and CI5791-γ8 gDNA enriched via exome capture on an Illumina NextSeq^®^ flow cell resulted in a total of 111,251,482, 103,796,564, and 120,530,567 reads, respectively. Thus, the parallel sequencing of the three exome-captured genotypes represented a balanced sequencing library. Utilizing the gene models underlying the ∼75 cM interval containing the mutant gene, deletion variant analysis identified a 1 bp cytosine deletion in the predicted coding region of the HORVU3Hr1G033740.2 gene model in CI5791-γ8 (Figure 3). Of the ∼120 million sequence reads from the CI5791-γ8 exome capture, 2,085 reads were aligned to the HORVU3Hr1G033740.2 gene model. The single base deletion in gene model HORVU3Hr1G033740.2 was the only deletion identified within the chromosome 3H region containing the mutant gene (Figure 2). The single base deletion in HORVU3Hr1G033740.2 is predicted to be within the second predicted exon, of the barley ortholog of the Arabidopsis WRKY transcription factor 6 gene, designated *HvWRKY6*. Coverage analysis showed that *HvWRKY6* is completely deleted from the CI5791-γ3 mutant as only three sequence reads from the CI5791-γ3 mutant were mapped to the HORVU3Hr1G033740.2 reference sequence gene model (Figure 3). Considering the balance of the multiplexed sequencing library which yielded 111,251,482, 103,796,564, and 120,530,567 reads for CI5791 WT, CI5791-γ3, and CI5791-γ8, respectively, and the numbers of reads that mapped to *HvWRKY6* for CI5791 WT (1,983) and CI5791-γ8 (2,085), the three reads that mapped to CI5791-γ3 is well below the threshold of contamination (Figure 3).

### Characterization of the Candidate Gene

**FIGURE 5:**
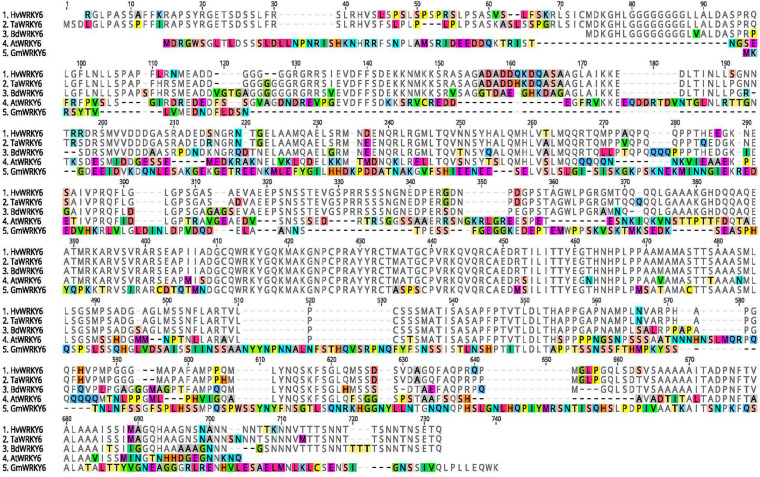
Protein sequence alignment of barley (*Hv*), wheat (*Ta*), *Brachypodium distachyon* (*Bd), Arabidopsis thaliana* (*At*), and soybean (*Gm*). Amino acids are highlighted when in disagreement with the consensus sequence. All five sequences and hyphens represent indels. Protein alignments were generated with Clustal Omega 1.2.2 (Sievers et al., 2011) plugin of Geneious Prime 2020.2.2 (https://www.geneious.com).

The variant analysis of the exome capture data pinpointed a single cytosine base deletion at nucleotide position 545 in relation to the adenosine of the start methionine as base 1 of the barley *HvWRKY6* gene model (Figure 3). The mutation in the CI5791-γ8 mutant occurs in the second exon, which resulted in a frame shift and the predicted translation of a non-functional 141 amino acid (aa) truncated protein (Figure 3). Read depth analysis showed that *HvWRKY6* was completely deleted from the CI5791-γ3 mutant as only three reads out of ∼104 million mapped to the HORVU3Hr1G033740.2 gene model compared to 1,983/∼111 million for CI5791 WT and 2,085/∼120 million for CI5791-γ8 (Figure 3). The *HvWRKY6* gene spans 8,026 bp of gDNA localized to barley chromosome 3H at ∼50.03 cM (Figure 2) based on POPSEQ positions (Mascher et al., 2013a). *HvWRKY6* is predicted to transcribe a 1,710 nucleotides mRNA consisting of six exons (Figure 3) predicted to encode a 569 aa functional protein (∼59.67 kDa) containing WRKY transcription factor domains including the highly conserved WRKYGQK DNA binding motif (Figures 3, 4). Homology searches utilizing NCBI BLASTp identified the candidate HORVU3Hr1G033740.2 predicted protein as an ortholog of the *Arabidopsis* WRKY transcription factor 6, thus, was designated *HvWRKY6*. The predicted HvWRKY6 protein has 50% aa identity and 59% aa similarity with the Arabidopsis WRKY6 protein (query cover 89% and *e*-value 4e^–130^) (Figure 5). A reciprocal result was obtained when the *AtWRKY6* protein was used as the query in a BLASTp search using the IPK barley blast server, identifying only one matching WRKY protein in the barley genome corresponding to HORVU3Hr1G033740.2. Thus, *HvWRKY6* represents the only known *AtWRKY6* ortholog in the barley genome. InterProScan SMART domain identified a conserved WRKY domain at 300-360 aa in *HvWRKY6* with high confidence prediction^2^ (Letunic et al., 2015). Analysis of the full length *HvWRKY6* gene sequence from CI5791, Morex, and Tifang were identical (Supplementary Figure 1) suggesting that the gene is conserved for its primary aa sequence across both resistant and susceptible barley genotypes. BLAST analysis of the barley pan-genome (Jayakodi et al., 2020) showed 100% sequence identity for the lines Akashinriki, Barke, Golden Promise, Hockett, HOR 3081, HOR 3365, HOR 7552, HOR 8148, HOR 9043, HOR 10350, HOR 13821, HOR 13942, HOR 22559, Igri, OUN333, RGT Planet, ZDM02064 (Jayakodi et al., 2020; Schreiber et al., 2020), Bowman (IBGSC, 2012), Haruna Nijo (Sato et al., 2016), and Lasa Goumang (Zeng et al., 2020). Whereas, ZDM01467 (Jayakodi et al., 2020) contained two concatenated hits of highly identical sequences suggesting a mis-assembly of the region. Zangqing320, a Tibeten hulless variety (Dai et al., 2018) and B1K-04-12, a wild barley (*H. vulgare* subsp. *spontaneum*, Jayakodi et al., 2020) both show 99.9% sequence identity. Two additional wild *hordeum* lines *H. bulbosum* and *H. pubiflorum* (Mascher et al., 2013b) showed, 96.8 and 95.4% sequence identity, respectively, further exemplifying the high conservation of HvWRKY6 in *hordeum*. In addition, *WRKY6* appears to be more highly conserved amongst monocots (wheat and *Brachypodium distachyyon*) than dicots (*Arabidopsis* and soybean, Figure 5).

### Validation of HvWRKY6 Function in CI5791 NFNB Resistance

The primer pair, WRKY6-F1 and WRKY6-Mt-R2, produced a CI5791-γ8 mutant specific *HvWRKY6* amplicon utilizing 3′-terminus specificity to the single nucleotide deletion based on the WRKY6-Mt-R2 primer sequence. The CI5791-γ8 mutant specific *HvWRKY6* primers did not produce an amplicon from the gDNA of WT CI5791, Heartland, or CI5791-γ3 (complete gene deletion) (Figure 3). The wild-type specific primer pair (WRKY6-F1 + WRKY6-Wt-R1) produced *HvWRKY6* specific amplicons from WT CI5791 and Heartland and did not produce amplicons from CI5791-γ3 or CI5791-γ8 mutants (Figure 3).

All homozygous susceptible F_2_ individuals from both the CI5791-γ3 × Heartland and CI5791-γ8 × Heartland populations showed a mutant *HvWRKY6* genotype with CI5791-γ8 mutant specific primers (WRKY6-F1 + WRKY6-Mt-R2; Figure 3) or WT specific primers. This was determined by no observed amplification with either primer pair on the CI5791-γ3 × Heartland homozygous susceptible F_2_ individuals, which is consistent with the entire gene deletion detected with the exome capture experiment (Figure 3). With the CI5791-γ8 × Heartland homozygous susceptible F_2_ individuals there were amplicons produced with the mutant specific primer pair (WRKY6-F1 + WRKY6-Mt-R2) but no observed amplification with the WT specific primer pair, which is consistent with the 1 bp deletion detected with the exome capture experiment showing that they were homozygous mutant individuals (Figure 3). Fifteen randomly selected resistant F_2_ individuals from the CI5791-γ8 × Heartland showed a 1 homozygous: 2 heterozygous genotype segregation (data not shown). This genotyping perfectly linked the genetic mutation with the mutant phenotype in this small F_2_ population representing 68 recombinant gametes. Also, the genotypes of all the reciprocal F_1_ (CI5791-γ3 × -γ8 or CI5791-γ8 × -γ3) individuals had a CI5791-γ8 mutant like genotype, lacking a WT allele, providing further evidence that the two mutants CI5791-γ3 and CI5791-γ8 are allelic.

### BSMV-VIGS

To validate the function of *HvWRKY6* in NFNB resistance the barley stripe mosaic virus (BSMV) tripartite genome was utilized for post-transcriptional gene silencing constructs (Figure 4). The disease reactions of the BSMV-*WRKY6* inoculated plants targeted for the post-transcriptional gene silencing of *HvWRKY6* were significantly more susceptible than the BSMV-*pBS* virus inoculated controls at both 7 and 12 days post inoculation when inoculated with *Ptt* isolate 0–1 (Figure 4 and Supplementary Tables 4, 8, 9). The BSMV-*pBs* virus control inoculations did not show the shift from resistance toward susceptibility.

### qPCR and RNAseq

**FIGURE 6:**
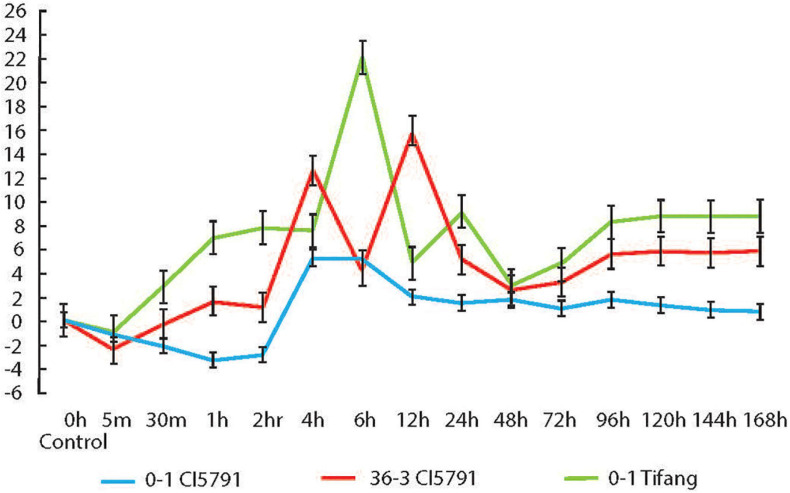
Relative expression of *HvWRKY6* in resistant CI5791 inoculated with *Pyrenophora teres* f. *teres* (*Ptt)* isolate 0–1 (blue) and moderately virulent isolate SM36-3 (red); and the susceptible cv. Tifang inoculated with *Ptt* isolate 0–1 (green). The *Y*-axis represents the fold change relative to the non-inoculated control (time point 0) and the *X*-axis represents the time point at which the leaf samples were collected.

The qPCR experiment conducted on WT CI5791 inoculated with the *Ptt* isolate 0–1 showed that *HvWRKY6* is upregulated at 4 h post inoculation (hpi) at least 5-fold until 6 hpi, then it gradually decreases and maintains a level of ∼1-fold upregulation until 168 hpi (Figure 6). With the Moroccan *Ptt* isolate SM36-3, which is moderately virulent on CI5791, *HvWRKY6* was upregulated 1 hpi by 1.6-fold and increased to 12.6-fold upregulation at 4 hpi, 4-fold upregulation at 6 hpi, 16-fold at 12 hpi, and maintained at least 5-fold upregulation after 96 through 168 hpi (Figure 6). When susceptible cultivar Tifang was challenged with *Ptt* isolate 0-1, *HvWRKY6* was upregulated 2.8-fold at 30 min post inoculation to 22-fold at 6 hpi, 4.8-fold at 12 hpi, 9-fold at 24 hpi, and maintained at least 8-fold upregulation after 96 through 168 hpi (Figure 6). The susceptible line Tifang was utilized in this study due to the fact that the 3H locus encompassing HvWRKY6 (Figure 2) was identified as a resistance QTL in a CI5791 x Tifang cross (Koladia et al., 2017). The qPCR analyses confirmed that the expression of *HvWRKY6* in CI5791 was significantly higher with the moderately virulent *Ptt* isolate SM36-3 than the avirulent isolate 0–1 from 1 to 4 h, and 96 to 168 h. Similarly, the expression of *HvWRKY6* in the susceptible line Tifang challenged with the virulent *Ptt* isolate 0–1 was much higher and significantly different than SM36-3 and 0–1 on CI5791 at the times between 30 min through 2 and 96 h through 168 h.

RNA sequencing was utilized for comparative global transcriptomics between non-inoculated and LDN-inoculated CI5791 at 3, 21, and 45 h post-inoculation. An upregulation of *HvWRKY6* (HORVU3Hr1G033740) in the inoculated samples was observed at all of the aforementioned time points (Supplementary Table 10) with *HvWRKY6* upregulated 7. 9-, 4. 6-, and 8.3-fold at 3, 21, and 45 h post-inoculation, respectively, in the CI5791 resistance response. As expected the gene enrichment analysis of differentially expressed genes at all the time points showed a significant enrichment of several classes of genes that are involved in the response to fungal pathogens (Supplementary Table 11).

## Discussion

Allelic deletion mutations that compromise NFNB resistance in barley line CI5791 were genetically mapped to an approximate 75 cM region of barley chromosome 3H. An exome capture mapping by sequencing approach identified independent mutations of the barley ortholog of the Arabidopsis *WRKY6* TF, designated *HvWRKY6*, in the delimited region. A 1 bp and whole *HvWRKY6* gene deletion in the two mutant lines CI5791-γ3 and CI5791-γ8 are consistent with the reports that γ-irradiation induce 1–10 kb deletions (Morita et al., 2009). The *HvWRKY6* gene was validated as playing a role in *Ptt* resistance via virus-induced gene silencing (VIGS) utilizing the barley stripe mosaic virus. Thus, we hypothesize that the *HvWRKY6* TF plays an important role in defense signaling that results in NFNB resistance.

The two independent mutants CI5791-γ3 and CI5791-γ8 exhibited susceptible symptoms to *Ptt* isolates that were not typical NFNB symptoms, but rather resemble SFNB lesions when inoculated with the *Ptt* isolates LDN and 0–1. The symptoms exhibited by the mutants were dark brown elliptical necrotic lesions surrounded by an extensive expanding yellow chlorotic margin (Figure 1A). The chlorosis expands and eventually coalesces with other lesions suggesting underlying pathogen growth, yet the necrotic regions remain relatively confined and elliptical, resembling an SFNB type of susceptible reaction (Figures 1A,B). Therefore, we initially phenotyped the CI5791-γ3 x Heartland F_2_ population using a 1-5 SFNB rating scale as described in Neupane et al. (2015). However, the CI5791-γ8 × Heartland F_2_ population, BSMV-VIGS experiment, and CI5791-γ3/CI5791-γ8 reciprocal allelism crosses were phenotyped at the F_1_ stage using the NFNB 1–10 rating scale as described by Tekauz (1985) as this mutant compromises NFNB disease resistance yet results in SFNB-like symptoms. Interestingly, the mutant’s symptoms when inoculated with the two Moroccan *Ptt* isolates, SM36-2 and SM36-3, resembled typical net type lesions with enlarged chlorosis that coalesced and has visible longitudinal and vertical striation but not as prominent as seen in a typical NFNB susceptible reaction. Thus, we speculate that the *HvWRKY6* transcription factor underlying the 3H QTL may function in regulating genes involved in restricting the growth of the pathogen. In a typical CI5791 resistance reaction the pathogen apparently penetrates the host as indicated by the formation of the pin point lesions, yet the pathogen growth is arrested early in the infection process and the lesion growth is effectively stopped at this early stage in the infection process. The CI5791 major dominant resistance gene responsible for this early resistance mechanism maps to the centromeric region of chromosome 6H (Koladia et al., 2017), which was the initial gene targeted in the mutant screening and was suspected to represent an immunity receptor. However, the first two mutants identified were allelic and mapped to the 3H QTL identified in the CI5791 x Tifang biparental mapping by Koladia et al. (2017) (Figure 2; Koladia et al., 2017) and disrupted the *HvWRKY6* transcription factor that plays a role in arresting pathogen colonization and spread after penetration. Additional studies have also reported large QLT intervals over chromosome 3H that spanned from 36.26 to 76.56 cM (Dinglasan et al., 2019). However, a smaller interval (51.27–51.77 cM) was consistently reported and designated as *qPttCLS*, that maps physically to 398.2–435.5 Mbp on the Morex reference genome (Mascher et al., 2017) and therefore is not suspected to encompass *HvWRKY6*. Several QTLs have been mapped to the 3H region, one at 28.7–36.6 cM (Steffenson et al., 1996), *QTLUHs-3H* at 29–31 cM (König et al., 2014), *QTL_*UH*_-3H* at 45–51 cM (König et al., 2013), and *Rpt-3H-4* at 57.0–66.6 cM (Yun et al., 2005) that may all be in close proximity, although these cannot be directly compared due to differing populations and absence of common marker sets. The locus, *NBP_QRptt3-2*, identified using GWAS spans 49.65-52.03 cM using POPSEQ positions and physically maps to 160.7–491.8 Mbp (Wonneberger et al., 2017) and therefore includes *HvWRKY6*. Additional studies have also reported significant markers in close physical and genetic proximity to *HvWRKY6* (Burlakoti et al., 2017; Islamovic et al., 2017; Koladia et al., 2017; Daba et al., 2019; Novakazi et al., 2019; Rozanova et al., 2019; Vatter et al., 2017).

Necrotrophic pathogens often produce several host specific necrotrophic effectors (NE) including low molecular weight metabolites and small secreted proteins that interact with dominant host susceptibility genes (Wolpert et al., 2002; Liu et al., 2011, 2012, 2015; Stergiopoulos et al., 2013; Shjerve et al., 2014). These interactions often follow the inverse-gene-for-gene model (Friesen et al., 2007) triggering programmed cell death (PCD) to facilitate necrotrophic fungal growth resulting in compatible interactions or a susceptible reaction called necrotrophic-effector triggered susceptibility (NETS) (Faris et al., 2010; Friesen and Faris, 2010; Liu et al., 2015; Faris and Friesen, 2020). *Ptt* is a necrotrophic pathogen that has been shown to produce a proteinaceous effector designated as PttNE1 that targets dominant susceptibility gene/s on chromosome 6H in barley (Liu et al., 2015) in an inverse gene-for-gene manner resulting in NETS. However, the CI5791 dominant resistance mechanism appears to follow the gene-for-gene model and possibly represents an R-gene-Avr gene interaction that results in an early dominant resistance response. The *HvWRKY6* gene appears to be a highly conserved TF that is most likely required for regulating early defense response genes post recognition that function to arrest pathogen spread after penetration. Thus, we hypothesize that it may be activated early in the response to the pathogen providing early resistance, which translates into preventing further proliferation of the fungus after penetration and thereby limiting the growth of the lesions. *Ptt* isolates SM36-2 and SM36-3 may express other effectors involved in virulence or have a variable Avr gene that evades early recognition and therefore activation of the early resistance signaling pathway, resulting in a moderately susceptible reaction in CI5791. Yet, this more prolific early pathogen growth and the chlorosis results in a higher level of susceptibility in the *HvWRKY6* mutants than in the WT, suggesting that the *HvWRKY6* transcription factor plays a role in regulating the early defense response genes that function to sequester pathogen spread after penetration thus inhibiting pathogen establishment in the host.

The qPCR analysis was performed because there appeared to be no polymorphism in the primary aa sequence of the *HvWRKY6* protein from a small number of resistant and susceptible genotypes analyzed, and BLAST analysis of 25 barley genotypes sequenced as part of a barley pan-genome project also showed 100% aa sequence identity for all but two of the *Hordeum vulgare* accessions sequenced suggesting that functional polymorphism between incompatible (resistant) and compatible (susceptible) interactions may be due to differential transcription. The qPCR analysis of HvWRKY6 was performed during infection processing. The qPCR data showed that the differential expression of *HvWRKY6* occurs in the barley line CI5791 in response to pathogen challenge as early as 1 hpi with the moderately virulent *Ptt* isolates SM36-3 and 4 hpi with the avirulent *Ptt* isolate 0-1. Similarly, in the susceptible line Tifang, the differential expression occurred as early as 30 min after inoculation and reached a maximum of 22-fold upregulation at 6 hpi. The line Tifang was utilized as the susceptible line in these analyses due to its use in the CI5791 x Tifang biparental population used to map the 3H QTL. Overall, the expression level in the susceptible cultivar Tifang with *Ptt* isolate 0-1 was significantly higher than CI5791 with 0-1 and SM36-3. The BSMV-VIGS experiment showed that the specific silencing of the candidate *HvWRKY6* gene results in a susceptible phenotype when inoculated with *Ptt* isolate 0-1. The time course RNA sequencing between non-inoculated and *Ptt* isolate LDN inoculated CI5791 seedlings showed upregulation of *HvWRKY6* in all the inoculated samples at all the time points (Supplementary Table 10). The gene enrichment analysis of differentially expressed genes at all the time points showed a significant enrichment and upregulation of several classes of genes that are involved in response to fungal pathogens including chitin responsive genes, salicylic acid (SA) and jasmonic acid (JA) response genes, and interestingly the upregulation of positive regulators of leaf senescence genes, which could play a role in suppressing the inverse gene-for-gene induction of PCD by this necrotrophic pathogen to facilitate disease (Supplementary Table 11).

The WRKY TFs are one of the largest groups of plant transcription regulators with protein domain architecture consisting of a highly conserved amino acid sequence (WRKYGQK) at the N-terminus and a zinc-finger-motif (C-C-H-H/C) at the C-terminus (Eulgem et al., 2000; Eulgem, 2006; Bakshi and Oelmüller, 2014). WRKY proteins bind specific W-box elements with the consensus sequence (TTGAC/T) at the promotor regions of targeted genes, resulting in transcriptional activation, and for some genes, repression (Eulgem et al., 2000; Yu et al., 2001; Rushton et al., 2010; Agarwal et al., 2011). WRKY TFs are important in diverse plant physiological activities such as pathogen defense responses and abiotic stress responses such as wounding, nutrient deficiency, salt stress (Chen et al., 2009; Kasajima et al., 2010; Cai et al., 2017; Hichri et al., 2017; Li et al., 2017), and developmental processes including senescence, and root growth (Robatzek and Somssich, 2001, 2002; Skibbe et al., 2008).

The *AtWRKY6* gene regulates both plant defense responses against *Pseudomonas syringae* pv. *tomato* as well as senescence in *Arabidopsis* (Robatzek and Somssich, 2002). *WRKY6* and *WRKY3* were also shown to regulate defense response in *Nicotiania attenuate* against the larvae of the insect herbivore *Manduca sexta* (Skibbe et al., 2008). In wheat, the *TaWRKY70* TF underlying the fusarium head blight QTL, *QTL-2DL*, governs resistance against *Fusarium graminearum* by regulating the downstream genes, *TaACT*, *TaDGK*, and *TaGLI*, which are involved in resistance responses (Kage et al., 2017). Other WRKY gene families have also been reported to play vital roles in defense responses in rice against *Magnaporthe grisea* (rice blast) and *Xanthomonas oryzea* (bacterial leaf blight) (Liu et al., 2007; Shimono et al., 2007; Wang et al., 2007). Li et al. (2004) reported enhanced resistance to the biotrophic fungal pathogen *Erysiphe cichoracearum*, whereas an increase in susceptibility to the bacterial necrotroph *Erwinia carotovora* subsp. *carotovora* occurred upon the upregulation of *WRKY70* in *Arabidopsis*.

The WRKY TFs also negatively regulate plant defense responses (Robatzek and Somssich, 2002; Eulgem and Somssich, 2007) as the overexpression of *WRKY38* and/or *WRKY62* were found to compromise immunity to the bacterial pathogen *P. syringae* and appeared to be negative regulators of plant basal defense responses (Mao et al., 2007; Kim et al., 2008). Grunewald et al. (2008) identified *WRKY23* as the negative regulator of plant defense responses against the cyst nematode *Heterodera schachtii.* The *WRKY11* and *WRKY17* TFs were also shown to function as negative regulators of basal defense responses in *Arabidopsis* (Journot-Catalino et al., 2006).

The data generated in this study show that the *HvWRKY6* gene functions in NFNB resistance and likely plays a role in the activation of defense genes that are required to restrict lesion growth once the pathogen attempts to penetrate or after it has entered the host. This question will be answered once microscopy analysis is performed on the mutant vs. wild-type CI5791 across the infection process. The *HvWRKY6* gene is expressed at higher levels during later time points in compatible interactions showing that the upregulation of the TF does not correlate with resistance. However, earlier upregulation of *HvWRKY6* in the incompatible interaction may be the key to resistance, suggesting that the TF may mediate a defense response that is only effective when induced early in the host-pathogen interaction. Also, the later induced expression of *HvWRKY6* is not deterministic of resistance as this later upregulation also occurs in the compatible interactions which actually had higher induced levels of *HvWRKY6* expression across most of the time points tested (Figure 6). Interestingly, some transcription factors do act as a negative regulator of the plant basal defense response when highly expressed, which has been shown in previous studies (Li et al., 2004; Journot-Catalino et al., 2006; Mao et al., 2007; Kim et al., 2008; Xing et al., 2008). However, the loss of *Ptt* resistance in the mutants suggested a positive role of *HvWRKY6* as the disruption of the gene produces a predicted non-functional protein in one mutant and the complete deletion of the gene in the other showing a predominantly positive role of *HvWRKY6* in the NFNB resistance responses.

Interestingly, the *HvWRKY6* gene falls directly under the CI5791 dominant resistance QTL identified by Koladia et al. (2017). However, allele analysis of Morex, CI5791, and Tifang alleles did not reveal any primary functional polymorphism (Supplementary Figure 1) and the expression analysis data did not convincingly determine any expression polymorphism that would explain the differential resistances and susceptibilities. Thus, it is likely that the *HvWRKY6* TF is involved in a basal resistance and may not represent the gene that underlies the 3H dominant resistance QTL reported by Koladia et al. (2017) as there was little information explaining functional polymorphism in a CI5791 × Tifang population. However, the possibility cannot be ruled out that slight expression differences from a finely tuned level between genotypes could result in the mapping of the resistance/susceptibility QTL on chromosome 3H. Polymorphisms could exist within additional regulatory regions that result in altered expression levels between the resistant and susceptible host genotypes and represent the functional polymorphisms that were segregated in the CI5791 × Tifang population. The fact that all currently analyzed barley lines are predicted to be translationally identical but have diverse phenotypic responses suggest that an upstream signaling component that upregulates *HvWRKY6* to a threshold for this resistance that if exceeded or not induced at all results in susceptibility. However, further genetic and functional analyses will be required to answer these questions.

Several studies have provided evidence that WRKY TFs are an integral part of the plant immune system including roles in PAMP-triggered immunity, effector-triggered immunity, and systemic acquired resistance (Li et al., 2004; Eulgem and Somssich, 2007; Rushton et al., 2010). Certain WRKY DNA-binding factors serve as components of signal transduction pathways in plant cells in response to pathogens and regulate the expression of certain plant defense genes (Riechmann et al., 2000). *AtWRKY6* regulates both plant defense responses against *P. syringae* pv. *tomato* as well as senescence in *Arabidopsis*, which regulates the *SIRK* gene (Senescence-Induced Receptor like serine/threonine protein Kinase) that encodes a receptor-like kinase that is exclusively localized to the plant cell nucleus (Robatzek and Somssich, 2002). The qPCR and RNAseq data reported here determined that *HvWRKY6* was upregulated in response to pathogen challenge and similar to *Arabidopsis* may regulate senescence-induced genes as this class of genes was shown to also be upregulated in response to the pathogen in the highly resistant barley line CI5791. Since the *TaWRKY70* TF was identified as a strong candidate gene that regulates *TaACT*, *TaDGK*, and *TaGLI* in wheat resistance to *Fusarium graminearum* (Kage et al., 2017), we hypothesize that *HvWRKY6* may regulate other defense related genes that are required to restrict pathogen/lesion growth in line CI5791.

Technological advances in genomic tools and methodology allows for the more accurate characterization of complex traits including quantitative disease resistances. Resistance or lack of susceptibility to the necrotrophic specialist pathogens like *P. teres* are typically quantitative in nature and the genes underlying the resistance loci have been difficult to localize and isolate. Utilizing forward genetics, mapping by sequencing, exome capture, and next generation sequencing data, we identified the *HvWRKY6* gene located under a chromosome 3H resistance QTL that is required for the broad and remarkable NFNB resistance in barley line CI5791. To the best of our knowledge this is the first resistance gene or component required for NFNB resistance to be identified. We hypothesize that the *HvWRKY6* transcription factor positively functions to regulate defense response genes, which are required for resistance to *Ptt* in the barley line CI5791 by limiting the growth of the pathogen in the host after initial entry. The work here exemplifies the powerful new molecular tools that will help generate knowledge and resources to genetically improve crops, through marker assisted and genomic selection strategies in a more intelligent manner. Thus, expediting agricultural productivity in a sustainable manner.

## Data Availability Statement

The exome capture data is publicly available and has been deposited on NCBI under the BioProject ID PRJNA686994. The RNAseq data has been deposited under the BioProject ID PRJNA683717. Further inquires can be directed to the corresponding author.

## Author Contributions

RB, TF, RH, and AB designed the experiments. XL and JH generated the mutant population. PT identified the mutants. PT, JR, SS, GA, PD, SC, and KE performed the wet lab experiments and analysis. JR and RS carried out the bioinformatics analysis. RB, PT, SC, KE, and AB wrote the manuscript. TF, JR, SS, SC, and GA proofread the manuscript. RB and AB obtained funding for research. All authors read and approved the final manuscript.

## Conflict of Interest

The authors declare that the research was conducted in the absence of any commercial or financial relationships that could be construed as a potential conflict of interest.

## Footnotes

1 http://webblast.ipk-gatersleben.de/barley_ibsc/

2 http://smart.embl.de/

## References

[B1] Abu Qamar M. Liu Z. H. Faris J. D. Chao S. Edwards M. C. Lai Z. (2008). A region of barley chromosome 6H harbors multiple major genes associated with net type net blotch resistance. *Theor. Appl. Genet.* 117 1261–1270. 10.1007/s00122-008-0860-x 18712341

[B2] Agarwal P. Reddy M. P. Chikara J. (2011). WRKY: its structure, evolutionary relationship, DNA-binding selectivity, role in stress tolerance and development of plants. *Mol. Biol. Rep.* 38 3883–3896. 10.1007/s11033-010-0504-5 21107718

[B3] Akhavan A. Turkington T. K. Askarian H. Tekauz A. Xi K. Tucker J. R. (2016). Virulence of *Pyrenophora teres* populations in western Canada. *Can. J. Plant Pathol.* 38 183–196. 10.1080/07060661.2016.1159617

[B4] Andolfo G. Jupe F. Witek K. Etherington G. J. Ercolano M. R. Jones J. D. G. (2014). Defining the full tomato NB-LRR resistance gene repertoire using genomic and cDNA RenSeq. *BMC Plant Biol.* 14:120. 10.1186/1471-2229-14-120 24885638 PMC4036795

[B5] Arabi M. I. Barrault G. Sarrafi A. Albertini L. (1992). Variation in the resistance of barley cultivars and in the pathogenicity of *Drechslera teres* f. sp. maculata and *D. teres* f. sp. teres isolates from France. *Plant Pathol.* 41 180–186. 10.1111/j.1365-3059.1992.tb02336.x

[B6] Arora S. Steuernagel B. Gaurav K. Chandramohan S. Long Y. Matny O. (2019). Resistance gene cloning from a wild crop relative by sequence capture and association genetics. *Nat. Biotechnol.* 37 139–143. 10.1038/s41587-018-0007-9 30718880

[B7] Bakshi M. Oelmüller R. (2014). WRKY transcription factors: jack of many trades in plants. *Plant Signal. Behav.* 9:e27700. 10.4161/psb.27700 24492469 PMC4091213

[B8] Bamshad M. J. Ng S. B. Bigham A. W. Tabor H. K. Emond M. J. Nickerson D. A. (2011). Exome sequencing as a tool for mendelian disease gene discovery. *Nat. Rev. Genet.* 12 745–755. 10.1038/nrg3031 21946919

[B9] Burlakoti R. R. Gyawali S. Chao S. Smith K. P. Horsley R. D. Cooper B. (2017). Genome-wide association study of spot form of net blotch resistance in the Upper Midwest barley breeding programs. *Phytopathology* 107 100–108. 10.1094/PHYTO-03-16-0136-R 27552325

[B10] Cai R. Dai W. Zhang C. Wang Y. Wu M. Zhao Y. (2017). The maize WRKY transcription factor ZmWRKY17 negatively regulates salt stress tolerance in transgenic *Arabidopsis* plants. *Planta* 246 1215–1231. 10.1007/s00425-017-2766-9 28861611

[B11] Cakir M. Gupta S. Platz G. J. Ablett G. A. Loughman R. Emebiri L. C. (2003). Mapping and validation of the genes for resistance to *Pyrenophora teres* f. *teres* in barley (*Hordeum vulgare* L.). *Aust. J. Agric. Res.* 54 1369–1377. 10.1071/AR02229

[B12] Chen Y.-F. Li L.-Q. Xu Q. Kong Y.-H. Wang H. Wu W.-H. (2009). The WRKY6 transcription factor modulates PHOSPHATE1 expression in response to low Pi stress in *Arabidopsis*. *Plant Cell* 21 3554–3566. 10.1105/tpc.108.064980 19934380 PMC2798333

[B13] Choi M. Scholl U. I. Ji W. Liu T. Tikhonova I. R. Zumbo P. (2009). Genetic diagnosis by whole exome capture and massively parallel DNA sequencing. *Proc. Natl. Acad. Sci. U.S.A.* 106 19096–19101. 10.1073/pnas.0910672106 19861545 PMC2768590

[B14] Clare S. J. Wyatt N. A. Brueggeman R. S. Friesen T. L. (2020). Research advances in the *Pyrenophora teres*–barley interaction. *Mol. Plant Pathol.* 21 272–288. 10.1111/mpp.12896 31837102 PMC6988421

[B15] Cosart T. Beja-Pereira A. Chen S. Ng S. B. Shendure J. Luikart G. (2011). Exome-wide DNA capture and next generation sequencing in domestic and wild species. *BMC Genomics* 12:347. 10.1186/1471-2164-12-347 21729323 PMC3146453

[B16] Daba S. D. Horsley R. Brueggeman R. Chao S. Mohammadi M. (2019). Genome-wide association studies and candidate gene identification for leaf scald and net blotch in barley (*Hordeum vulgare* L.). *Plant Dis.* 103 880–889. 10.1094/PDIS-07-18-1190-RE 30806577

[B17] Dai F. Wang X. Zhang X.-Q. Chen Z. Nevo E. Jin G. (2018). Assembly and analysis of a qingke reference genome demonstrate its close genetic relation to modern cultivated barley. *Plant Biotechnol. J.* 16 760–770. 10.1111/pbi.12826 28871634 PMC5814578

[B18] Dinglasan E. Hickey L. Ziems L. Fowler R. Anisimova A. Baranova O. (2019). Genetic characterization of resistance to *Pyrenophora teres* f. *teres* in the international barley differential canadian lake shore. *Front. Plant Sci.* 10:326. 10.3389/fpls.2019.00326 30967885 PMC6442539

[B19] Eulgem T. (2006). Dissecting the WRKY Web of plant defense regulators. *PLoS Pathog.* 2:e126. 10.1371/journal.ppat.0020126 17121464 PMC1657070

[B20] Eulgem T. Rushton P. J. Robatzek S. Somssich I. E. (2000). The WRKY superfamily of plant transcription factors. *Trends Plant Sci.* 5 199–206. 10.1016/S1360-1385(00)01600-9 10785665

[B21] Eulgem T. Somssich I. E. (2007). Networks of WRKY transcription factors in defense signaling. *Curr. Opin. Plant Biol.* 10 366–371. 10.1016/j.pbi.2007.04.020 17644023

[B22] Faris J. D. Friesen T. L. (2020). Plant genes hijacked by necrotrophic fungal pathogens. *Curr. Opin. Plant Biol.* 56 74–80. 10.1016/j.pbi.2020.04.003 32492572

[B23] Faris J. D. Zhang Z. Lu H. Lu S. Reddy L. Cloutier S. (2010). A unique wheat disease resistance-like gene governs effector-triggered susceptibility to necrotrophic pathogens. *Proc. Natl. Acad. Sci. U.S.A.* 107 13544–13549. 10.1073/pnas.1004090107 20624958 PMC2922177

[B24] Ferdous J. Li Y. Reid N. Langridge P. Shi B.-J. Tricker P. J. (2015). Identification of reference genes for quantitative expression analysis of microRNAs and mRNAs in barley under various stress conditions. *PLoS One* 10:e0118503. 10.1371/journal.pone.0118503 25793505 PMC4368757

[B25] Friesen T. L. Faris J. D. (2010). Characterization of the wheat-Stagonospora nodorum disease system: what is the molecular basis of this quantitative necrotrophic disease interaction?†. *Can. J. Plant Pathol.* 32 20–28. 10.1080/07060661003620896

[B26] Friesen T. L. Faris J. D. Lai Z. Steffenson B. J. (2006). Identification and chromosomal location of major genes for resistance to *Pyrenophora teres* in a doubled-haploid barley population. *Genome* 49 855–859. 10.1139/g06-024 16936794

[B27] Friesen T. L. Meinhardt S. W. Faris J. D. (2007). The *Stagonospora nodorum*-wheat pathosystem involves multiple proteinaceous host-selective toxins and corresponding host sensitivity genes that interact in an inverse gene-for-gene manner. *Plant J.* 51 681–692. 10.1111/j.1365-313X.2007.03166.x 17573802

[B28] Grewal T. S. Rossnagel B. G. Pozniak C. J. Scoles G. J. (2008). Mapping quantitative trait loci associated with barley net blotch resistance. *Theor. Appl. Genet.* 116 529–539. 10.1007/s00122-007-0688-9 18071668

[B29] Grunewald W. Karimi M. Wieczorek K. Van de Cappelle E. Wischnitzki E. Grundler F. (2008). A role for AtWRKY23 in feeding site establishment of plant-parasitic nematodes. *Plant Physiol.* 148 358–368. 10.1104/pp.108.119131 18599655 PMC2528098

[B30] Heffelfinger C. Fragoso C. A. Lorieux M. (2017). Constructing linkage maps in the genomics era with MapDisto 2.0. *Bioinformatics* 33 2224–2225. 10.1093/bioinformatics/btx177 28369214 PMC5870660

[B31] Hichri I. Muhovski Y. Žižková E. Dobrev P. I. Gharbi E. Franco-Zorrilla J. M. (2017). The Solanum lycopersicum WRKY3 Transcription Factor slwrky3 is involved in salt stress tolerance in tomato. *Front. Plant Sci.* 8:1343. 10.3389/fpls.2017.01343 28824679 PMC5534461

[B32] Horsley R. Franckowiak J. Schwarz P. (2009). “Barley,” in *Cereals. Handbook of Plant Breeding*, Vol. 3 ed. Carena M. (New York, NY: Springer), 10.1007/978-0-387-72297-9_7

[B33] IBGSC (2012). A physical, genetic and functional sequence assembly of the barley genome. *Nature* 491:711. 10.1038/nature11543 23075845

[B34] Islamovic E. Bregitzer P. Friesen T. L. (2017). Barley 4H QTL confers NFNB resistance to a global set of *P. teres* f. *teres* isolates. *Mol. Breed.* 37:29 10.1007/s11032-017-0621-0

[B35] Jayakodi M. Padmarasu S. Haberer G. Bonthala V. S. Gundlach H. Monat C. (2020). The barley pan-genome reveals the hidden legacy of mutation breeding. *Nature* 588, 284–289. 10.1038/s41586-020-2947-8 33239781 PMC7759462

[B36] Joehanes R. Nelson J. C. (2008). QGene 4.0, an extensible Java QTL-analysis platform. *Bioinformatics* 24 2788–2789. 10.1093/bioinformatics/btn523 18940826

[B37] Journot-Catalino N. Somssich I. E. Roby D. Kroj T. (2006). The Transcription Factors WRKY11 and WRKY17 act as negative regulators of basal resistance in &lt;em&gt;*Arabidopsis thaliana*&lt;/em&gt. *Plant Cell* 18 3289–3302. 10.1105/tpc.106.044149 17114354 PMC1693958

[B38] Jupe F. Witek K. Verweij W. Śliwka J. Pritchard L. Etherington G. J. (2013). Resistance gene enrichment sequencing (RenSeq) enables reannotation of the NB-LRR gene family from sequenced plant genomes and rapid mapping of resistance loci in segregating populations. *Plant J.* 76 530–544. 10.1111/tpj.12307 23937694 PMC3935411

[B39] Kage U. Yogendra K. N. Kushalappa A. C. (2017). TaWRKY70 transcription factor in wheat QTL-2DL regulates downstream metabolite biosynthetic genes to resist Fusarium graminearum infection spread within spike. *Sci. Rep.* 7:42596. 10.1038/srep42596 28198421 PMC5309853

[B40] Kasajima I. Ide Y. Yokota Hirai M. Fujiwara T. (2010). WRKY6 is involved in the response to boron deficiency in *Arabidopsis thaliana*. *Physiol. Plant.* 139 80–92. 10.1111/j.1399-3054.2010.01349.x 20059736

[B41] Kim K.-C. Lai Z. Fan B. Chen Z. (2008). *Arabidopsis* WRKY38 and WRKY62 transcription factors interact with histone deacetylase 19 in basal defense. *Plant Cell* 20 2357–2371. 10.1105/tpc.107.055566 18776063 PMC2570728

[B42] Koladia V. M. Faris J. D. Richards J. K. Brueggeman R. S. Chao S. Friesen T. L. (2017). Genetic analysis of net form net blotch resistance in barley lines CIho 5791 and Tifang against a global collection of P. teres f. teres isolates. *Theor. Appl. Genet.* 130 163–173. 10.1007/s00122-016-2801-4 27734097

[B43] König J. Perovic D. Kopahnke D. Ordon F. (2013). Development of an efficient method for assessing resistance to the net type of net blotch (*Pyrenophora teres* f. *teres*) in winter barley and mapping of quantitative trait loci for resistance. *Mol. Breed.* 32 641–650. 10.1007/s11032-013-9897-x

[B44] König J. Perovic D. Kopahnke D. Ordon F. (2014). Mapping seedling resistance to net form of net blotch (*Pyrenophora teres* f. *teres*) in barley using detached leaf assay. *Plant Breed* 133 356–365.

[B45] Letunic I. Doerks T. Bork P. (2015). SMART: recent updates, new developments and status in 2015. *Nucleic Acids Res.* 43 D257–D260. 10.1093/nar/gku949 25300481 PMC4384020

[B46] Li J. Brader G. Palva E. T. (2004). The WRKY70 transcription factor: a node of convergence for jasmonate-mediated and salicylate-mediated signals in plant defense. *Plant Cell* 16 319–331. 10.1105/tpc.016980 14742872 PMC341906

[B47] Li L.-Q. Huang L.-P. Pan G. Liu L. Wang X.-Y. Lu L.-M. (2017). Identifying the genes regulated by AtWRKY6 using comparative transcript and proteomic analysis under phosphorus deficiency. *Int. J. Mol. Sci.* 18:1046. 10.3390/ijms18051046 28498313 PMC5454958

[B48] Liu X. Bai X. Wang X. Chu C. (2007). OsWRKY71, a rice transcription factor, is involved in rice defense response. *J. Plant Physiol.* 164 969–979. 10.1016/j.jplph.2006.07.006 16919842

[B49] Liu Z. Ellwood S. R. Oliver R. P. Friesen T. L. (2011). *Pyrenophora teres*: profile of an increasingly damaging barley pathogen. *Mol. Plant Pathol.* 12 1–19. 10.1111/j.1364-3703.2010.00649.x 21118345 PMC6640222

[B50] Liu Z. Holmes D. J. Faris J. D. Chao S. Brueggeman R. S. Edwards M. C. (2015). Necrotrophic effector-triggered susceptibility (NETS) underlies the barley–*Pyrenophora teres* f. *teres* interaction specific to chromosome 6H. *Mol. Plant Pathol.* 16 188–200. 10.1111/mpp.12172 25040207 PMC6638325

[B51] Liu Z. H. Zhong S. Stasko A. K. Edwards M. C. Friesen T. L. (2012). Virulence profile and genetic structure of a North Dakota population of *Pyrenophora teres* f. teres, the causal agent of net form net blotch of Barley. *Phytopathology* 102 539–546. 10.1094/PHYTO-09-11-0243 22494251

[B52] Mao P. Duan M. Wei C. Li Y. (2007). WRKY62 transcription factor acts downstream of cytosolic NPR1 and negatively regulates jasmonate-responsive gene expression. *Plant Cell Physiol.* 48 833–842. 10.1093/pcp/pcm058 17510065

[B53] Mascher M. Gundlach H. Himmelbach A. Beier S. Twardziok S. O. Wicker T. (2017). A chromosome conformation capture ordered sequence of the barley genome. *Nature* 544 427–433. 10.1038/nature22043 28447635

[B54] Mascher M. Jost M. Kuon J.-E. Himmelbach A. Aßfalg A. Beier S. (2014). Mapping-by-sequencing accelerates forward genetics in barley. *Genome Biol.* 15:R78. 10.1186/gb-2014-15-6-r78 24917130 PMC4073093

[B55] Mascher M. Muehlbauer G. J. Rokhsar D. S. Chapman J. Schmutz J. Barry K. (2013a). Anchoring and ordering NGS contig assemblies by population sequencing (POPSEQ). *Plant J.* 76 718–727. 10.1111/tpj.12319 23998490 PMC4298792

[B56] Mascher M. Richmond T. A. Gerhardt D. J. Himmelbach A. Clissold L. Sampath D. (2013b). Barley whole exome capture: a tool for genomic research in the genus Hordeum and beyond. *Plant J.* 76 494–505. 10.1111/tpj.12294 23889683 PMC4241023

[B57] Mascher M. Schuenemann V. J. Davidovich U. Marom N. Himmelbach A. Hübner S. (2016). Genomic analysis of 6,000-year-old cultivated grain illuminates the domestication history of barley. *Nat. Genet.* 48:1089. 10.1038/ng.3611 27428749

[B58] Mathre D. E. (1997). *Compendium of Barley Diseases*, 2nd Edn St. Paul, Minnesota: APS Press.

[B59] Mode C. J. Schaller C. W. (1958). Two additional factors for host resistance to net blotch in barley. *Agron. J.* 50 15–18.

[B60] Morita R. Kusaba M. Iida S. Yamaguchi H. Nishio T. Nishimura M. (2009). Molecular characterization of mutations induced by gamma irradiation in rice. *Genes Genet. Syst.* 84 361–370. 10.1266/ggs.84.361 20154423

[B61] Murray G. M. Brennan J. P. (2010). Estimating disease losses to the Australian barley industry. *Australas. Plant Pathol.* 39 85–96. 10.1071/AP09064

[B62] Neupane A. Tamang P. Brueggeman R. S. Friesen T. L. (2015). Evaluation of a barley core collection for spot form net blotch reaction reveals distinct genotype-specific pathogen virulence and host susceptibility. *Phytopathology* 105 509–517. 10.1094/PHYTO-04-14-0107-R 25870926

[B63] Novakazi F. Afanasenko O. Anisimova A. Platz G. J. J. Snowdon R. Kovaleva O. (2019). Genetic analysis of a worldwide barley collection for resistance to net form of net blotch disease (*Pyrenophora teres* f. *teres*). *Theor. Appl. Genet.* 132 2633–2650. 10.1007/s00122-019-03378-1 31209538

[B64] Raca G. Jackson C. Warman B. Bair T. Schimmenti L. A. (2010). Next generation sequencing in research and diagnostics of ocular birth defects. *Mol. Genet. Metab.* 100 184–192. 10.1016/j.ymgme.2010.03.004 20359920 PMC2871986

[B65] Richards J. Chao S. Friesen T. Brueggeman R. (2016). Fine mapping of the barley chromosome 6H net form net blotch susceptibility locus. *G3 Genes| Genomes| Genetics* 6 1809–1818. 27172206 10.1534/g3.116.028902 PMC4938636

[B66] Richards J. K. K. Friesen T. L. L. Brueggeman R. S. S. (2017). Association mapping utilizing diverse barley lines reveals net form net blotch seedling resistance/susceptibility loci. *Theor. Appl. Genet.* 130 915–927. 10.1007/s00122-017-2860-1 28184981

[B67] Riechmann J. L. Heard J. Martin G. Reuber L. Jiang C.-Z. Keddie J. (2000). *Arabidopsis transcription factors: genome-wide comparative analysis among eukaryotes*. *Science* 290 2105L–2110. 10.1126/science.290.5499.2105 11118137

[B68] Robatzek S. Somssich I. E. (2001). A new member of the *Arabidopsis* WRKY transcription factor family, AtWRKY6, is associated with both senescence- and defence-related processes. *Plant J.* 28 123–133. 10.1046/j.1365-313X.2001.01131.x 11722756

[B69] Robatzek S. Somssich I. E. (2002). Targets of AtWRKY6 regulation during plant senescence and pathogen defense. *Genes Dev.* 16 1139–1149. 10.1101/gad.222702 12000796 PMC186251

[B70] Rozanova I. V. Lashina N. M. Mustafin Z. S. Gorobets S. A. Efimov V. M. Afanasenko O. S. (2019). SNPs associated with barley resistance to isolates of *Pyrenophora teres* f. *teres*. *BMC Genomics* 20:292. 10.1186/s12864-019-5623-3 32039701 PMC7227216

[B71] Rushton P. J. Somssich I. E. Ringler P. Shen Q. J. (2010). WRKY transcription factors. *Trends Plant Sci.* 15 247–258. 10.1016/j.tplants.2010.02.006 20304701

[B72] Russell J. Mascher M. Dawson I. K. Kyriakidis S. Calixto C. Freund F. (2016). Exome sequencing of geographically diverse barley landraces and wild relatives gives insights into environmental adaptation. *Nat. Genet.* 48:1024. 10.1038/ng.3612 27428750

[B73] Sato K. Tanaka T. Shigenobu S. Motoi Y. Wu J. Itoh T. (2016). Improvement of barley genome annotations by deciphering the Haruna Nijo genome. *DNA Res.* 23 21–28. 10.1093/dnares/dsv033 26622062 PMC4755524

[B74] Schreiber M. Barakate A. Uzrek N. Macaulay M. Sourdille A. Morris J. (2019). A highly mutagenised barley (cv. Golden Promise) TILLING population coupled with strategies for screening-by-sequencing. *Plant Methods* 15:99. 10.1186/s13007-019-0486-9 31462905 PMC6708184

[B75] Schreiber M. Mascher M. Wright J. Padmarasu S. Himmelbach A. Heavens D. (2020). A genome assembly of the barley ‘Transformation Reference’ cultivar golden promise. *G3 (Bethesda)* 10 1823–1827. 10.1101/2020.02.12.945550 32241919 PMC7263683

[B76] Shimono M. Sugano S. Nakayama A. Jiang C.-J. Ono K. Toki S. (2007). Rice WRKY45 plays a crucial role in benzothiadiazole-inducible blast resistance. *Plant Cell* 19 2064–2076. 10.1105/tpc.106.046250 17601827 PMC1955718

[B77] Shjerve R. A. Faris J. D. Brueggeman R. S. Yan C. Zhu Y. Koladia V. (2014). Evaluation of a *Pyrenophora teres* f. *teres* mapping population reveals multiple independent interactions with a region of barley chromosome 6H. *Fungal Genet. Biol.* 70 104–112. 10.1016/j.fgb.2014.07.012 25093269

[B78] Sievers F. Wilm A. Dineen D. G. Gibson T. J. Karplus K. Li W. (2011). Fast, scalable generation of high-quality protein multiple sequence alignments using Clustal Omega. *Mol. Syst. Biol.* 7:539. 10.1038/msb.2011.75 21988835 PMC3261699

[B79] Skibbe M. Qu N. Galis I. Baldwin I. T. (2008). Induced plant defenses in the natural environment: Nicotiana attenuata WRKY3 and WRKY6 coordinate responses to herbivory. *Plant Cell* 20 1984–2000. 10.1105/tpc.108.058594 18641266 PMC2518244

[B80] Smedegård-Petersen V. (1971). *Pyrenophora teres f. maculata* f. nov. and *Pyrenophora tere*s *f. teres* on barley in Denmark. *Kgl. Vet. Landbohojsk. Arsskr* 124–144.

[B81] Solanki S. Richards J. Ameen G. Wang X. Khan A. Ali H. (2019). Characterization of genes required for both *Rpg1* and *rpg4*-mediated wheat stem rust resistance in barley. *BMC Genomics* 20:495. 10.1186/s12864-019-5858-z. 31200635 PMC6570958

[B82] Steffenson B. J. Hayes P. M. Kleinhofs A. (1996). Genetics of seedling and adult plant resistance to net blotch (*Pyrenophora teres* f. *teres*) and spot blotch (*Cochliobolus sativus*) in barley. *Theor. Appl. Genet.* 92 552–558. 10.1007/BF00224557 24166322

[B83] Steffenson B. J. Webster R. K. (1992). Pathotype diversity of *Pyrenophora teres* f. *teres* on barley. *Phytopathology* 82 170–177. 10.1094/PHYTO.1999.89.2.176 18944793

[B84] Stergiopoulos I. Collemare J. Mehrabi R. De Wit P. J. G. M. (2013). Phytotoxic secondary metabolites and peptides produced by plant pathogenic Dothideomycete fungi. *FEMS Microbiol. Rev.* 37 67–93. 10.1111/j.1574-6976.2012.00349.x 22931103

[B85] Steuernagel B. Periyannan S. K. Hernández-Pinzón I. Witek K. Rouse M. N. Yu G. (2016). Rapid cloning of disease-resistance genes in plants using mutagenesis and sequence capture. *Nat. Biotechnol.* 34 652–655. 10.1038/nbt.3543 27111722

[B86] Tekauz A. (1985). A numerical scale to classify reactions of barley to *Pyrenophora teres*. *Can. J. Plant Pathol.* 7 181–183. 10.1080/07060668509501499

[B87] Therrien M. C. Wolfe R. I. (1985). Heartland Barley. *Can. J. Plant Sci.* 65 445–446. 10.4141/cjps85-060

[B88] Vatter T. Maurer A. Kopahnke D. Perovic D. Ordon F. Pillen K. (2017). A nested association mapping population identifies multiple small effect QTL conferring resistance against net blotch (*Pyrenophora teres* f. *teres*) in wild barley. *PLoS One* 12:e0186803. 10.1371/journal.pone.0186803 29073176 PMC5658061

[B89] Wang H. Chattopadhyay A. Li Z. Daines B. Li Y. Gao C. (2010). Rapid identification of heterozygous mutations in *Drosophila* melanogaster using genomic capture sequencing. *Genome Res.* 20 981–988. 10.1101/gr.102921.109 20472684 PMC2892099

[B90] Wang H. Hao J. Chen X. Hao Z. Wang X. Lou Y. (2007). Overexpression of rice WRKY89 enhances ultraviolet B tolerance and disease resistance in rice plants. *Plant Mol. Biol.* 65 799–815. 10.1007/s11103-007-9244-x 17960484

[B91] Warr A. Robert C. Hume D. Archibald A. Deeb N. Watson M. (2015). Exome sequencing: current and future perspectives. *G3 Genes Genomes Genetics* 5 1543–1550. 10.1534/g3.115.018564 26139844 PMC4528311

[B92] Weiland J. J. Steffenson B. J. Cartwright R. D. Webster R. K. (1999). Identification of molecular genetic markers in *Pyrenophora teres* f. teres associated with low virulence on ‘Harbin’ barley. *Phytopathology* 89 176–181. 10.1094/PHYTO.1999.89.2.176 18944793

[B93] Witek K. Jupe F. Witek A. I. Baker D. Clark M. D. Jones J. D. G. (2016). Accelerated cloning of a potato late blight–resistance gene using RenSeq and SMRT sequencing. *Nat. Biotechnol.* 34 656–660. 10.1038/nbt.3540 27111721

[B94] Wolpert T. J. Dunkle L. D. Ciuffetti L. M. (2002). Host-selective toxins and avirulence determinants: what’s in a name? *Annu. Rev. Phytopathol.* 40 251–285. 10.1146/annurev.phyto.40.011402.114210 12147761

[B95] Wonneberger R. Ficke A. Lillemo M. (2017). Mapping of quantitative trait loci associated with resistance to net form net blotch (*Pyrenophora teres* f. *teres*) in a doubled haploid Norwegian barley population. *PLoS One* 12:e0175773. 10.1371/journal.pone.0175773 28448537 PMC5407769

[B96] Wu H.-L. Steffenson B. J. Zhong S. Li Y. Oleson A. E. (2003). Genetic variation for virulence and RFLP markers in *Pyrenophora teres*. *Can. J. Plant Pathol.* 25 82–90. 10.1080/07060660309507052

[B97] Wyatt N. A. Richards J. K. Brueggeman R. S. Friesen T. L. (2018). Reference assembly and annotation of the *Pyrenophora teres* f. *teres* isolate 0-1. *G3 Genes| Genomes| Genetics* 8 1–8. 29167271 10.1534/g3.117.300196 PMC5765338

[B98] Xie W. Xiong W. Pan J. Ali T. Cui Q. Guan D. (2018). Decreases in global beer supply due to extreme drought and heat. *Nat. Plants* 4 964–973. 10.1038/s41477-018-0263-1 30323183

[B99] Xing D.-H. Lai Z.-B. Zheng Z.-Y. Vinod K. M. Fan B.-F. Chen Z.-X. (2008). Stress- and pathogen-induced *Arabidopsis* WRKY48 is a transcriptional activator that represses plant basal defense. *Mol. Plant* 1 459–470. 10.1093/mp/ssn020 19825553

[B100] Yu D. Chen C. Chen Z. (2001). Evidence for an important role of WRKY DNA binding proteins in the regulation of NPR1 gene expression. *Plant Cell* 13 1527–1540. 10.1105/tpc.010115 11449049 PMC139550

[B101] Yun S. J. Gyenis L. Hayes P. M. Matus I. Smith K. P. Steffenson B. J. (2005). Quantitative trait loci for multiple disease resistance in wild barley. *Crop Sci.* 45 2563–2572. 10.2135/cropsci2005.0236

[B102] Zeng X. Xu T. Ling Z. Wang Y. Li X. Xu S. (2020). An improved high-quality genome assembly and annotation of Tibetan hulless barley. *Sci. Data* 7:139. 10.1038/s41597-020-0480-0 32385314 PMC7210891

