# Supplementary material for: The Barley HvWRKY6 Transcription Factor Is Required for Resistance Against Pyrenophora teres f. teres
Source: Front Genet. 2021 Jan 15;11:601500. doi: 10.3389/fgene.2020.601500 (PMC7844392; doi:10.3389/fgene.2020.601500)
Supplement: Supplementary file 1 [file Table_1.docx]

**Supporting Information**

**Supporting Information Figure Legends**

**SI Figure 1.** Nucleotide alignment of *HvWRKY6* from three barley genotypes*.* The grey bars for barley genotypes Morex, CI5791 and Tifang show 100% nucleic acid conservation of the HvWRKY6 gene from genomic DNA alignments. The promoter and 5’ UTR is represented by the green arrow, exons by red arrows, introns by grey arrows and 3’ UTR by the blue arrow.

**Supporting Information Figure 1.**

**
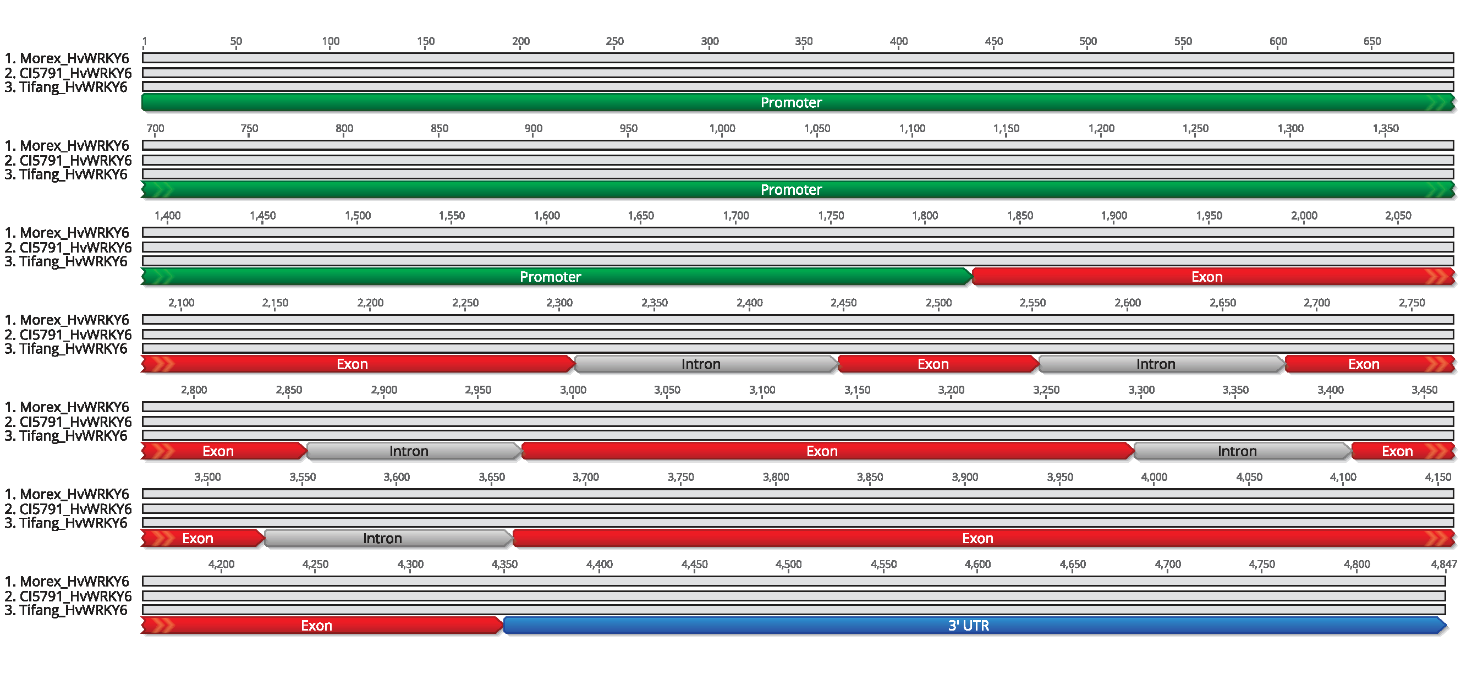
**

**SI Table 1.** Seedling disease reactions of 111 CI5791-γ3 X Heartland F_2_ individuals to *Ptt* isolate 0-1 based on the 1-5 SFNB rating scale. Disease reactions above 2 were considered susceptible.

| **SN** | **CI5791-γ3 X Heartland F2** | **Score** |
| --- | --- | --- |
| 1 | γ3/ HL-1 | 2.0 |
| 2 | γ3/ HL-2 | 1.0 |
| 3 | γ3/ HL-3 | 1.5 |
| 4 | γ3/ HL-4 | 1.0 |
| 5 | γ3/ HL-5 | 1.0 |
| 6 | γ3/ HL-6 | 1.0 |
| 7 | γ3/ HL-7 | 1.0 |
| 8 | γ3/ HL-8 | 2.0 |
| 9 | γ3/ HL-9 | 1.0 |
| 10 | γ3/ HL-10 | 1.0 |
| 11 | γ3/ HL-11 | 1.0 |
| 12 | γ3/ HL-12 | 1.5 |
| 13 | γ3/ HL-13 | 1.0 |
| 14 | γ3/ HL-14 | 1.5 |
| 15 | γ3/ HL-15 | 1.0 |
| 16 | γ3/ HL-16 | 1.5 |
| 17 | γ3/ HL-17 | 1.0 |
| 18 | γ3/ HL-18 | 1.0 |
| 19 | γ3/ HL-19 | 1.0 |
| 20 | γ3/ HL-20 | 1.0 |
| 21 | γ3/ HL-21 | 2.0 |
| 22 | γ3/ HL-22 | 1.0 |
| 23 | γ3/ HL-23 | 1.0 |
| 24 | γ3/ HL-24 | 1.0 |
| 25 | γ3/ HL-25 | 3.0 |
| 26 | γ3/ HL-26 | 3.5 |
| 27 | γ3/ HL-27 | 4.0 |
| 28 | γ3/ HL-28 | 4.0 |
| 29 | γ3/ HL-29 | 4.5 |
| 30 | γ3/ HL-30 | 4.0 |
| 31 | γ3/ HL-31 | 4.5 |
| 32 | γ3/ HL-32 | 4.5 |
| 33 | γ3/ HL-33 | 4.5 |
| 34 | γ3/ HL-34 | 3.5 |
| 35 | γ3/ HL-35 | 3.5 |
| 36 | γ3/ HL-36 | 4.5 |
| 37 | γ3/ HL-38 | 3.5 |
| 38 | γ3/ HL-39 | 4.0 |
| 39 | γ3/ HL-40 | 4.0 |
| 40 | γ3/ HL-41 | 4.5 |
| 41 | γ3/ HL-42 | 4.0 |
| 42 | γ3/ HL-43 | 4.0 |
| 43 | γ3/ HL-44 | 4.5 |
| 44 | γ3/ HL-45 | 1.0 |
| 45 | γ3/ HL-46 | 1.0 |
| 46 | γ3/ HL-47 | 1.5 |
| 47 | γ3/ HL-48 | 1.0 |
| 48 | γ3/ HL-49 | 1.0 |
| 49 | γ3/ HL-50 | 1.0 |
| 50 | γ3/ HL-51 | 2.5 |
| 51 | γ3/ HL-1 | 1.0 |
| 52 | γ3/ HL-2 | 1.0 |
| 53 | γ3/ HL-3 | 2.0 |
| 54 | γ3/ HL-4 | 1.0 |
| 55 | γ3/ HL-5 | 1.0 |
| 56 | γ3/ HL-6 | 1.5 |
| 57 | γ3/ HL-7 | 1.0 |
| 58 | γ3/ HL-8 | 1.0 |
| 59 | γ3/ HL-9 | 1.0 |
| 60 | γ3/ HL-10 | 1.0 |
| 61 | γ3/ HL-11 | 1.5 |
| 62 | γ3/ HL-12 | 1.0 |
| 63 | γ3/ HL-13 | 1.0 |
| 64 | γ3/ HL-14 | 1.0 |
| 65 | γ3/ HL-15 | 1.0 |
| 66 | γ3/ HL-16 | 1.0 |
| 67 | γ3/ HL-17 | 1.0 |
| 68 | γ3/ HL-18 | 1.0 |
| 69 | γ3/ HL-19 | 1.0 |
| 70 | γ3/ HL-20 | 1.0 |
| 71 | γ3/ HL-21 | 1.0 |
| 72 | γ3/ HL-22 | 1.0 |
| 73 | γ3/ HL-23 | 1.0 |
| 74 | γ3/ HL-24 | 1.0 |
| 75 | γ3/ HL-25 | 1.0 |
| 76 | γ3/ HL-26 | 1.0 |
| 77 | γ3/ HL-27 | 1.0 |
| 78 | γ3/ HL-28 | 1.5 |
| 79 | γ3/ HL-29 | 1.0 |
| 80 | γ3/ HL-30 | 1.5 |
| 81 | γ3/ HL-31 | 1.0 |
| 82 | γ3/ HL-32 | 1.5 |
| 83 | γ3/ HL-33 | 1.5 |
| 84 | γ3/ HL-34 | 1.0 |
| 85 | γ3/ HL-35 | 1.0 |
| 86 | γ3/ HL-36 | 1.5 |
| 87 | γ3/ HL-37 | 1.0 |
| 88 | γ3/ HL-38 | 2.0 |
| 89 | γ3/ HL-40 | 3.5 |
| 90 | γ3/ HL-41 | 4.0 |
| 91 | γ3/ HL-42 | 4.0 |
| 92 | γ3/ HL-43 | 4.0 |
| 93 | γ3/ HL-44 | 3.5 |
| 94 | γ3/ HL-45 | 3.0 |
| 95 | γ3/ HL-46 | 3.5 |
| 96 | γ3/ HL-47 | 4.0 |
| 97 | γ3/ HL-48 | 5.0 |
| 98 | γ3/ HL-49 | 4.5 |
| 99 | γ3/ HL-50 | 4.0 |
| 100 | γ3/ HL-51 | 4.0 |
| 101 | γ3/ HL-52 | 1.0 |
| 102 | γ3/ HL-53 | 1.0 |
| 103 | γ3/ HL-54 | 1.0 |
| 104 | γ3/ HL-55 | 1.0 |
| 105 | γ3/ HL-56 | 1.0 |
| 106 | γ3/ HL-57 | 1.0 |
| 107 | γ3/ HL-58 | 1.0 |
| 108 | γ3/ HL-59 | 1.0 |
| 109 | γ3/ HL-60 | 1.0 |
| 110 | γ3/ HL-61 | 3.0 |
| 111 | γ3/ HL-62 | 3.5 |
| 112 | CI5791-γ3 | 3.5 |
| 113 | CI 5791 | 1.0 |
| 114 | Heartland | 1.0 |

**SI Table 2.** Primer sequences used to amplify the whole *HvWRKY6* gene for sequencing.

| Primers | Sequence (5'-3') |
| --- | --- |
| Wrky6-gen-F1 | GATGAGCATCCAGTAAGCTGTCACTG |
| Wrky6-gen-R1 | GACTAACAGGTGCAACGATGCG |
| Wrky6-gen-F2 | CGAGGAAGTTGGAGCTCATGAGC |
| Wrky6-gen-R2 | CTCCGACTCACGAGGTACTATTC |
| Wrky6-gen-F3 | CACGATGGCACTCTCGTTCTTG |
| Wrky6-gen-R3 | GGCAGCTTGGCTTCTTGAACTTG |
| Wrky6-gen-F4 | CTTCTTCTCGTCGGAGAAGAAGTC |
| Wrky6-gen-R4 | CTACTATTCCTCGTGCAGTACGTG |

**SI Table 3.** Seedling disease phenotype of reciprocal cross between CI5791-γ3 and CI5791-γ8 F_1_s to *Ptt* isolate 0-1.

| **SN** | **Plants** | **Disease score** |
| --- | --- | --- |
| 1 | CI5791-γ8/-γ3 - 1 | 6.5 |
| 2 | CI5791-γ8/-γ3 - 2 | 7.5 |
| 3 | CI5791-γ8/-γ3 - 3 | 7.0 |
| 4 | CI5791-γ8/-γ3 - 4 | 6.0 |
| 5 | CI5791-γ8/-γ3 - 5 | 5.5 |
| 6 | CI5791-γ8/-γ3 - 6 | 6.5 |
| 7 | CI5791-γ8/-γ3 - 7 | 5.5 |
| 8 | CI5791-γ8/-γ3 - 8 | 7.0 |
| 9 | CI5791-γ8/-γ3 - 9 | 7.0 |
| 10 | CI5791-γ8/-γ3 - 10 | 6.0 |
| 11 | CI5791-γ3/-γ8 - 1 | 5.5 |
| 12 | CI5791-γ3/-γ8 - 2 | 9.0 |
| 13 | CI5791-γ3/-γ8 - 3 | 5.5 |
| 14 | CI5791-γ3/-γ8 - 4 | 6.0 |
| 15 | CI5791-γ3/-γ8 - 5 | 6.0 |
| 16 | CI5791-γ3/-γ8 - 6 | 5.5 |
| 17 | CI5791-γ3 | 6.0 |
| 18 | CI5791-γ8 | 5.5 |
| 19 | CI5791 | 1.5 |
| 20 | Heartland | 1.5 |
| 21 | Robust | 8.5 |

**SI Table 4.** Phenotypic analysis of BSMBV-VIGS plants inoculated with *Ptt* isolate 0-1.

| **Days post inoculation (dpi)** | **VIGS Knockdown** | **VIGS Control** | **Pr > \|t\|** |
| --- | --- | --- | --- |
| 7 dpi | 4.67 ± 1.17 | 3.06 ± 1.09 | <.0001 |
| 12 dpi | 6.00 ± 1.79 | 2.99 ± 1.23 | <.0001 |

**SI Table 5.** Seedling disease reactions of 116 CI5791-γ8 X Heartland F_2_ individuals to *Ptt* isolate 0-1 based on the 1-10 NFNB rating scale. Disease reactions above 3 were considered susceptible.

| **SN** | CI5791-γ8 X Heartland | | **Score** | |  |
| --- | --- | --- | --- | --- | --- |
| 1 | γ8/ HL - 1 | | 6.0 | |  |
| 2 | γ8/ HL - 2 | | 7.0 | |  |
| 3 | γ8/ HL - 3 | | 6.5 | |  |
| 4 | γ8/ HL - 4 | | 6.0 | |  |
| 5 | γ8/ HL - 5 | | 5.0 | |  |
| 6 | γ8/ HL - 6 | | 5.5 | |  |
| 7 | γ8/ HL - 7 | | 8.0 | |  |
| 8 | γ8/ HL - 8 | | 6.0 | |  |
| 9 | γ8/ HL - 9 | | 6.5 | |  |
| 10 | γ8/ HL - 10 | | 7.5 | |  |
| 11 | γ8/ HL - 12 | | 4.5 | |  |
| 12 | γ8/ HL - 13 | | 6.0 | |  |
| 13 | γ8/ HL - 14 | | 6.5 | |  |
| 14 | γ8/ HL - 15 | | 7.0 | |  |
| 15 | γ8/ HL - 16 | | 8.5 | |  |
| 16 | γ8/ HL - 17 | | 6.5 | |  |
| 17 | γ8/ HL - 18 | | 9.0 | |  |
| 18 | γ8/ HL - 19 | | 7.5 | |  |
| 19 | γ8/ HL - 20 | | 6.5 | |  |
| 20 | γ8/ HL - 21 | | 7.0 | |  |
| 21 | γ8/ HL - 22 | | 5.5 | |  |
| 22 | γ8/ HL - 23 | | 8.0 | |  |
| 23 | γ8/ HL - 24 | | 6.5 | |  |
| 24 | γ8/ HL - 25 | | 6.0 | |  |
| 25 | γ8/ HL - 26 | | 6.0 | |  |
| 26 | γ8/ HL - 27 | | 7.5 | |  |
| 27 | γ8/ HL - 28 | | 7.5 | |  |
| 28 | γ8/ HL - 29 | | 2.0 | |  |
| 29 | γ8/ HL - 30 | | 1.0 | |  |
| 30 | γ8/ HL - 31 | | 1.5 | |  |
| 31 | γ8/ HL - 32 | | 1.0 | |  |
| 32 | γ8/ HL - 33 | | 1.5 | |  |
| 33 | γ8/ HL - 34 | | 1.5 | |  |
| 34 | γ8/ HL - 35 | | 1.0 | |  |
| 35 | γ8/ HL - 36 | | 1.5 | |  |
| 36 | γ8/ HL - 37 | | 1.5 | |  |
| 37 | γ8/ HL - 38 | | 1.0 | |  |
| 38 | γ8/ HL - 39 | | 1.5 | |  |
| 39 | γ8/ HL - 40 | | 1.5 | |  |
| 40 | γ8/ HL - 41 | | 1.5 | |  |
| 41 | γ8/ HL - 42 | | 1.5 | |  |
| 42 | | γ8/ HL - 43 | | 1.0 | |
| 43 | | γ8/ HL - 44 | | 2.5 | |
| 44 | | γ8/ HL - 45 | | 2.0 | |
| 45 | | γ8/ HL - 46 | | 2.0 | |
| 46 | | γ8/ HL - 47 | | 2.2 | |
| 47 | | γ8/ HL - 48 | | 1.0 | |
| 48 | | γ8/ HL - 49 | | 1.0 | |
| 49 | | γ8/ HL - 50 | | 1.0 | |
| 50 | | γ8/ HL - 51 | | 1.5 | |
| 51 | | γ8/ HL - 52 | | 1.0 | |
| 52 | | γ8/ HL - 53 | | 1.5 | |
| 53 | | γ8/ HL - 54 | | 1.0 | |
| 54 | | γ8/ HL - 55 | | 2.0 | |
| 55 | | γ8/ HL - 56 | | 1.5 | |
| 56 | | γ8/ HL - 57 | | 2.0 | |
| 57 | | γ8/ HL - 58 | | 1.5 | |
| 58 | | γ8/ HL - 59 | | 2.0 | |
| 59 | | γ8/ HL - 60 | | 1.5 | |
| 60 | | γ8/ HL - 61 | | 1.0 | |
| 61 | | γ8/ HL - 62 | | 1.0 | |
| 62 | | γ8/ HL - 63 | | 1.5 | |
| 63 | | γ8/ HL - 64 | | 2.5 | |
| 64 | | γ8/ HL - 65 | | 2.0 | |
| 65 | | γ8/ HL - 66 | | 1.0 | |
| 66 | | γ8/ HL - 67 | | 1.5 | |
| 67 | | γ8/ HL - 68 | | 1.0 | |
| 68 | | γ8/ HL - 69 | | 1.0 | |
| 69 | | γ8/ HL - 70 | | 1.5 | |
| 70 | | γ8/ HL - 71 | | 1.0 | |
| 71 | | γ8/ HL - 72 | | 1.0 | |
| 72 | | γ8/ HL - 73 | | 1.0 | |
| 73 | | γ8/ HL - 74 | | 1.0 | |
| 74 | | γ8/ HL - 75 | | 2.0 | |
| 75 | | γ8/ HL - 76 | | 1.5 | |
| 76 | | γ8/ HL - 77 | | 1.5 | |
| 77 | | γ8/ HL - 78 | | 1.0 | |
| 78 | | γ8/ HL - 79 | | 1.5 | |
| 79 | | γ8/ HL - 80 | | 1.5 | |
| 80 | | γ8/ HL - 81 | | 1.0 | |
| 81 | | γ8/ HL - 82 | | 2.0 | |
| 82 | | γ8/ HL - 83 | | 1.5 | |
| 83 | | γ8/ HL - 84 | 1.5 | |  |
| 85 | | γ8/ HL - 86 | | 1.0 | |
| 86 | | γ8/ HL - 87 | | 1.0 | |
| 87 | | γ8/ HL - 88 | | 1.0 | |
| 88 | | γ8/ HL - 89 | | 1.5 | |
| 89 | | γ8/ HL - 90 | | 1.0 | |
| 90 | | γ8/ HL - 91 | | 2.0 | |
| 91 | | γ8/ HL - 92 | | 2.5 | |
| 92 | | γ8/ HL - 93 | | 2.0 | |
| 93 | | γ8/ HL - 94 | | 1.5 | |
| 94 | | γ8/ HL - 95 | | 1.5 | |
| 95 | | γ8/ HL - 96 | | 2.0 | |
| 96 | | γ8/ HL - 97 | | 2.0 | |
| 97 | | γ8/ HL - 98 | | 1.5 | |
| 98 | | γ8/ HL - 99 | | 1.5 | |
| 99 | | γ8/ HL - 100 | | 1.5 | |
| 100 | | γ8/ HL - 101 | | 1.0 | |
| 101 | | γ8/ HL - 102 | | 1.0 | |
| 102 | | γ8/ HL - 103 | | 1.5 | |
| 103 | | γ8/ HL - 104 | | 1.0 | |
| 104 | | γ8/ HL - 105 | | 1.0 | |
| 105 | | γ8/ HL - 106 | | 2.0 | |
| 106 | | γ8/ HL - 107 | | 2.0 | |
| 107 | | γ8/ HL - 108 | | 2.0 | |
| 108 | | γ8/ HL - 109 | | 1.5 | |
| 109 | | γ8/ HL - 110 | | 1.5 | |
| 110 | | γ8/ HL - 111 | | 2.0 | |
| 111 | | γ8/ HL - 112 | | 1.0 | |
| 112 | | γ8/ HL - 113 | | 1.5 | |
| 113 | | γ8/ HL - 114 | | 1.5 | |
| 114 | | γ8/ HL - 115 | | 1.0 | |
| 115 | | γ8/ HL - 116 | | 1.5 | |
| 116 | | γ8/ HL - 117 | | 1.0 | |
| 117 | | CI5791-γ3 | | 6.5 | |
| 118 | | CI5791-γ8 | | 6.0 | |
| 119 | | Heartland | | 1.5 | |
| 120 | | CI5791 | | 1.5 | |
| 121 | | Robust | | 8.5 | |
| 122 | | Tifang | | 8.0 | |

**SI Table 6.** Seedling disease phenotype of CI5791-γ3, CI5791-γ8, CI5791, Hockett, and Hector to the *Ptt* isolates SM36-2 and SM36-3 collected in Morocco.

| **Lines** | ***Ptt* SM-36-2** | ***Ptt* SM36-3** |
| --- | --- | --- |
| CI5791-γ3 | 6.5 | 6.0 |
| CI5791-γ8 | 7.0 | 6.5 |
| CI5791 | 4.5 | 3.5 |
| Hockett | 1.0 | 1.0 |
| Hector | 9.5 | 8.0 |

**SI Table 7.** Segregation of CI5791-γ3 x Heartland and CI5791-γ8 x Heartland F_2_ individuals inoculated with *Ptt* isolate 0-1.

| **Populations** | **Resistant F_2_** | **Susceptible F_2_** | **χ^2^ (3:1)^*^** |
| --- | --- | --- | --- |
| CI5791-γ3 F_2_s | 77 | 34 | 1.87 |
| CI5791-γ8 F_2_s | 89 | 27 | 0.26 |

*Non-significant at *p*=0.05 level.

**SI Table 8.** Phenotypic analysis of BSMV-VIGS plants inoculated with *Ptt* isolate 0-1 at 7 DAI (days after inoculation).

| **Plants** | **VIGS Knockdown** | **Plants** | **MCS** |
| --- | --- | --- | --- |
| 1 | 5.3 | 1 | 2.3 |
| 2 | 5.3 | 2 | 2.0 |
| 3 | 5.3 | 3 | 5.3 |
| 4 | 2.0 | 4 | 3.3 |
| 5 | 4.3 | 5 | 2.3 |
| 6 | 4.3 | 6 | 2.0 |
| 7 | 5.0 | 7 | 2.0 |
| 8 | 6.0 | 8 | 2.3 |
| 9 | 6.8 | 9 | 4.8 |
| 10 | 5.8 | 10 | 2.3 |
| 11 | 3.3 | 11 | 2.0 |
| 12 | 5.0 | 12 | 4.3 |
| 13 | 4.3 | 13 | 3.0 |
| 14 | 2.3 | 14 | 2.8 |
| 15 | 5.3 | 15 | 3.3 |
| 16 | 4.3 | 16 | 2.3 |
| 17 | 4.8 | 17 | 4.3 |
| 18 | 3.0 | 18 | 3.0 |
| 19 | 5.3 | 19 | 4.3 |
| 20 | 2.3 | 20 | 5.0 |
| 21 | 4.8 | 21 | 2.0 |
| 22 | 6.0 | 22 | 4.0 |
| 23 | 5.0 | 23 | 3.0 |
| 24 | 3.3 | 24 | 5.0 |
| 25 | 6.0 | 25 | 3.8 |
| 26 | 5.8 | 26 | 3.0 |
| 27 | 6.3 | 27 | 2.3 |
| 28 | 5.0 | 28 | 2.3 |
| 29 | 4.3 | 29 | 3.3 |
| 30 | 3.0 | 30 | 2.0 |
| 31 | 6.0 | 31 | 2.8 |
| 32 | 3.8 | 32 | 2.8 |
| 33 | 5.0 | 33 | 2.8 |
| 34 | 6.0 | CI 5791 | 1.3 |
| 35 | 4.8 | Robust | 8.0 |
| 36 | 5.0 |  |  |
| 37 | 5.0 |  |  |
| 38 | 3.3 |  |  |
| 39 | 4.8 |  |  |

**SI Table 9.** Phenotypic analysis of BSMV-VIGS plants inoculated with *Ptt* isolate 0-1 at 12 DAI (days after inoculation).

| **Plants** | **VIGS Knockdown** | **Plants** | **MCS** |
| --- | --- | --- | --- |
| 1 | 5.0 | 1 | 1.0 |
| 2 | 5.0 | 2 | 2.0 |
| 3 | 7.3 | 3 | 2.0 |
| 4 | 6.3 | 4 | 2.0 |
| 5 | 4.3 | 5 | 1.3 |
| 6 | 3.8 | 6 | 4.3 |
| 7 | 6.3 | 7 | 2.8 |
| 8 | 4.8 | 8 | 2.3 |
| 9 | 3.3 | 9 | 2.3 |
| 10 | 5.3 | 10 | 2.3 |
| 11 | 8.3 | 11 | 2.3 |
| 12 | 5.3 | 12 | 2.3 |
| 13 | 7.3 | 13 | 2.3 |
| 14 | 10.0 | 14 | 5.3 |
| 15 | 9.0 | 15 | 3.3 |
| 16 | 8.3 | 16 | 4.3 |
| 17 | 9.0 | 17 | 2.3 |
| 18 | 7.3 | 18 | 4.3 |
| 19 | 3.3 | 19 | 4.3 |
| 20 | 4.3 | 20 | 3.0 |
| 21 | 5.0 | 21 | - |
| 22 | 3.3 | 22 | 4.3 |
| 23 | 6.8 | 23 | 3.3 |
| 24 | 3.3 | 24 | 3.3 |
| 25 | 7.3 | 25 | 5.0 |
| 26 | 5.0 | 26 | 2.0 |
| 27 | 7.0 | 27 | 2.0 |
| 28 | 4.3 | 28 | 2.3 |
| 29 | 5.3 | 29 | 6.3 |
| 30 | 6.3 | 30 | 2.8 |
| 31 | 7.3 | 31 | 3.3 |
| 32 | 5.3 | 32 | 4.3 |
| 33 | 7.3 | 33 | 2.0 |
| 34 | 7.3 | CI 5791 | 1.3 |
| 35 | 7.0 | Robust | 9.5 |

**SI Table 10**. Fold change of *HvWrky6* in pathogen inoculated samples at different time points compared to its non-inoculated control

| Comparison | **Gene** | **Fold Change^a^** | **FDR-Pvalue^b^** |
| --- | --- | --- | --- |
| Control vs 3HPI | HORVU3Hr1G033740 | 7.842921 | 9.3E-11 |
| Control vs 21HPI | HORVU3Hr1G033740 | 5.176195 | 6.33E-07 |
| Control vs 45HPI | HORVU3Hr1G033740 | 7.958703 | 8.3E-16 |

a- A Bioconductor packaged EDGE embedded in CLC genomics workbench v8.0 was used to calculated fold changed based on RPKM (Read per kilobases per million) value

b- False discovery rate (FDR) corrected p-value (level of significant, FDR p-value < 0.05)

**SI Table 11**.

Significantly enriched Gene ontology terms in pathogen inoculated samples at different time points compared to the non-inoculated samples

| DEG^a^ | GO ID^b^ | GO Term^c^ |
| --- | --- | --- |
| Upregulated | GO:0006468 | protein phosphorylation |
|  | GO:0009611 | response to wounding |
|  | GO:0010200 | response to chitin |
|  | GO:0055114 | oxidation-reduction process |
|  | GO:0000162 | tryptophan biosynthetic process |
|  | GO:0080167 | response to karrikin |
|  | GO:0042742 | defense response to bacterium |
|  | GO:0009651 | response to salt stress |
|  | GO:0007166 | cell surface receptor signaling pathway |
|  | GO:0006979 | response to oxidative stress |
|  | GO:0031408 | oxylipin biosynthetic process |
|  | GO:0009751 | response to salicylic acid |
|  | GO:0009753 | response to jasmonic acid |
|  | GO:0046323 | glucose import |
|  | GO:0046686 | response to cadmium ion |
|  | GO:0009409 | response to cold |
|  | GO:0009407 | toxin catabolic process |
|  | GO:1900056 | negative regulation of leaf senescence |
|  | GO:0080148 | negative regulation of response to water deprivation |
|  | GO:0042744 | hydrogen peroxide catabolic process |
|  | GO:0006032 | chitin catabolic process |
|  | GO:0048544 | recognition of pollen |
|  | GO:0009809 | lignin biosynthetic process |
| Downregulated | GO:0055114 | oxidation-reduction process |
|  | GO:0009768 | photosynthesis, light harvesting in photosystem I |
|  | GO:0018298 | protein-chromophore linkage |
|  | GO:0009409 | response to cold |
|  | GO:0009414 | response to water deprivation |
|  | GO:0009645 | response to low light intensity stimulus |
|  | GO:0034220 | ion transmembrane transport |
|  | GO:0010114 | response to red light |
|  | GO:0009644 | response to high light intensity |
|  | GO:0015995 | chlorophyll biosynthetic process |
|  | GO:0010218 | response to far red light |
|  | GO:0009737 | response to abscisic acid |
|  | GO:0006833 | water transport |
|  | GO:0009903 | chloroplast avoidance movement |
|  | GO:0009611 | response to wounding |
|  | GO:0009635 | response to herbicide |
|  | GO:0006814 | sodium ion transport |
|  | GO:0009769 | photosynthesis, light harvesting in photosystem II |
|  | GO:0080027 | response to herbivore |
|  | GO:0080167 | response to karrikin |
|  | GO:0008152 | metabolic process |

a-Differentially expressed genes (DEG) that are upregulated or downregulated in *Ptt* inoculated samples compared to non-inoculated samples.

b- GO IDs that are common in all comparison (Control vs 3HPI, Control vs 21HPI and Control vs 45HPI) for a given upregulated or downregulated set of genes

The GO term enrichment analysis was done in the bioconductor R package TopGO version 2.28.0 (Alexa and Rahnenfuhrer, 2010)

Alexa, A. and Rahnenfuhrer, J., 2010. topGO: enrichment analysis for gene ontology. *R package version*, *2*(0).
